# Extended sister-chromosome catenation leads to massive reorganization of the *E. coli* genome

**DOI:** 10.1093/nar/gkac105

**Published:** 2022-02-25

**Authors:** Brenna Conin, Ingrid Billault-Chaumartin, Hafez El Sayyed, Nicole Quenech’Du, Charlotte Cockram, Romain Koszul, Olivier Espéli

**Affiliations:** Center for Interdisciplinary Research in Biology (CIRB), Collége de France, CNRS, INSERM, Université PSL, Paris, France; Institut Pasteur, Université de Paris, CNRS UMR3525, Unité Régulation Spatiale des Génomes, F-75015 Paris, France; Collège Doctoral, Sorbonne Université, F-75005 Paris, France; Center for Interdisciplinary Research in Biology (CIRB), Collége de France, CNRS, INSERM, Université PSL, Paris, France; Center for Interdisciplinary Research in Biology (CIRB), Collége de France, CNRS, INSERM, Université PSL, Paris, France; Center for Interdisciplinary Research in Biology (CIRB), Collége de France, CNRS, INSERM, Université PSL, Paris, France; Institut Pasteur, Université de Paris, CNRS UMR3525, Unité Régulation Spatiale des Génomes, F-75015 Paris, France; Institut Pasteur, Université de Paris, CNRS UMR3525, Unité Régulation Spatiale des Génomes, F-75015 Paris, France; Center for Interdisciplinary Research in Biology (CIRB), Collége de France, CNRS, INSERM, Université PSL, Paris, France

## Abstract

In bacteria, chromosome segregation occurs progressively from the origin to terminus within minutes of replication of each locus. Between replication and segregation, sister loci are held in an apparent cohesive state by topological links. The decatenation activity of topoisomerase IV (Topo IV) is required for segregation of replicated loci, yet little is known about the structuring of the chromosome maintained in a cohesive state. In this work, we investigated chromosome folding in cells with altered decatenation activities. Within minutes after Topo IV inactivation, massive chromosome reorganization occurs, associated with increased in contacts between nearby loci, likely trans-contacts between sister chromatids, and in long-range contacts between the terminus and distant loci. We deciphered the respective roles of Topo III, MatP and MukB when TopoIV activity becomes limiting. Topo III reduces short-range inter-sister contacts suggesting its activity near replication forks. MatP, the terminus macrodomain organizing system, and MukB, the *Escherichia coli* SMC, promote long-range contacts with the terminus. We propose that the large-scale conformational changes observed under these conditions reveal defective decatenation attempts involving the terminus area. Our results support a model of spatial and temporal partitioning of the tasks required for sister chromosome segregation.

## INTRODUCTION

Prokaryotic and eukaryotic chromosomes are not randomly folded, but consist of well-defined structural entities with a complex hierarchical organization. The regulation of this network and its functional interplay with gene expression or other chromosomal metabolic processes such as DNA repair, replication and segregation have been actively investigated in a number of species (1). Improvement in imaging techniques of living cells, as well as the development of genomic approaches such as chromosome conformation capture (3C/Hi-C3) techniques have highlighted a mosaic of intertwined structural features including loops, domains, and compartments (2).

In bacteria, an important part of genome folding relies on the presence of free DNA supercoils. Independent supercoiling domains were first observed in the 1970s through a combination of molecular biology and electron microscopy approaches (3,4). These textbook pictures consist of a succession of large (∼200 kb) DNA plectonemes, or microdomains, that are delimited by topological insulators. Genomic and recombination assays confirmed the existence of these microdomains, but found that they are likely smaller in size (10–50 kb), delimited by stochastic barriers (5,6). These results suggest that microdomains are not static, and that their size and position can be modulated by DNA transactions such as transcription (7) or the binding of proteins to DNA (8). The democratization of 3C/Hi-C methodologies provided the opportunity to further assess the impact of DNA supercoiling on chromosome folding. To date, 3C/Hi-C data revealed the organization of large chromosomal features, such as the alignment of replication arms (9–11), macrodomains (12) or inter-chromosomal contacts (13). At smaller scale, Hi-C contact maps of every bacterial genome studied to date display self-interacting domains (CIDs, 20–200 kb), whose significance remain unclear (9–14). The boundaries in-between CIDs correlate with the presence of long, highly expressed genes or genes coding membrane proteins (9,12,15).

Experiments and simulations suggest that supercoils are able to organize bacterial genome because they would condense DNA and promote the disentanglement of topological domains(16) (. DNA supercoiling is under tight homeostatic control by topoisomerases. In *Escherichia coli*, Topoisomerase I (Topo I), Topoisomerase III (Topo III), DNA Gyrase and Topoisomerase IV (Topo IV) have all well characterized enzymatic activities (17,18), but understanding the roles played by topoisomerases in chromosomal compaction, folding and organization is still a work in progress. DNA Gyrase, that promotes the formation of free supercoils, is the best candidate for regulation of chromosome organization through supercoiling. Hi-C analysis following gyrase inhibition was studied in *Caulobacter crescentus*, revealing modest changes in chromosome conformation with a slight decrease of 20- to 200-kb contacts that reduced the sharpness and positions of CID boundaries (9). This observation agrees with recombination data showing that topoisomerase alterations reduce supercoiling domain's size (19). The contribution of other topoisomerases in establishing, maintaining, and regulating genome-wide DNA contacts has, so far, not been investigated.

The *E. coli* SMC complex MukBEF was shown to be linked to DNA supercoiling, with MukBEF defects being suppressed by Topo I mutation (20). The suppression correlates with an excess of negative supercoiling promoted by DNA gyrase. In addition, Hi-C analysis of a MukB mutant showed an important loss of long-range contacts (12), suggesting that MukB promotes contacts between distant pairs of loci. One hypothesis is that MukB could promote DNA loops *in vivo* (21–24). For instance, it is possible that MukB, organised in an axial core, extrudes plectonemic microdomains (25). Interestingly, MukB interacts with ParC, the catalytic subunit of Topo IV. Their interaction changes the catalytic properties of Topo IV (26,27) and modulates its localization (28,29). *In vitro* assays suggest that a MukB – Topo IV interaction promotes DNA compaction by forcing the intramolecular knotting activity of Topo IV (30). Single molecule imaging reveals that a small portion of ParC molecules are associated with MukB in small clusters, (29). The absence of MukB leads to a 2-fold reduction of Topo IV clusters, suggesting that MukB influences the localization of Topo IV in the cell

Topo IV is the main bacterial decatenase. It catalyzes the elimination of precatenanes and catenanes formed during replication (31), and is essential for proper chromosome segregation (32–35). The requirement of PriA, the main replication fork restart protein, for the survival of Topo IV thermosensitive mutants (*parE^ts^* and *parC^ts^*), suggests that Topo IV inactivation increases replication fork stalling (36). Nevertheless, chromosome replication in *parE^ts^* mutant cells occurs at a similar rate to that seen in *wt* cells (35). Topo IV activity is highest at the *dif* site positioned in the middle of the Terminus of replication macrodomain, presumably to solve catenanes (37–39). However, it also presents hundreds of putative activity sites dispersed on the genome, presumably used for the removal of precatenanes (37).

Here, we investigate, using Hi-C, the effect of different topoisomerases on *E. coli* chromosome conformation. Inactivation of Topo IV generates the most significant changes in chromosome organization, with a pair of long-range contact patterns, hereafter referred to as butterfly wings, expanding from the *terminus* macrodomain flanking regions. Imaging experiments confirmed the chromosome reorganization when Topo IV activity is reduced, suggesting that under these conditions distant sister loci segregate when they contact the terminus. Because MatP and MukB also influence the butterfly motif, we propose that it reveals a particular genome folding dedicated to decatenation. We also showed that when Topo IV is deficient, Hi-C reveals inter-molecular contacts between sister chromosomes. Topo III limits these contacts suggesting that it removes a significant portion of precatenanes. Together, our results highlight the ability of Hi-C to unveil chromosomal topological features, including inter-chromosomal contacts, to improve our understanding of the mechanisms governing chromosome segregation.

## MATERIALS AND METHODS

**Strains:** The strains used for this study can be found in Supplementary Table 1. All strains are derived from MG1655. All strains were grown in minimal media A (0.26 M KH_2_PO_4_, 0.06 M K_2_HPO_4_, 0.01 M tri sodium citrate, 2 mM MgSO_4_, 0.04 M (NH4)_2_SO_4_) supplemented with 0.2% of casamino acids and 0.5% of glucose. The strains containing the thermosensitive allele of *parC* (*parC^ts^*) were grown at 25°C and shifted to 30°C for 60 min, which is semi-permissive temperature; the strains containing the thermosensitive allele of *parE* (*parE^ts^*) were grown at 30°C and shifted at 42°C for 60 min at most. Time course of *parE^ts^* shift was performed by growing the cells at 30°C before performing a shift for 10, 20, 30, 40, 50 and 60 min. Strains containing the expression plasmid pBAD were cultivated with 0.2% arabinose to induce the expression of the gene under the control of the PBAD promoter the arabinose was present for the entire duration of the culture. For each imaging or Hi-C experiment a *wt* MG1655 strain was grown in the exact conditions as the mutant and used as reference for subsequent comparisons.

**Drugs and antibiotics:** Inhibition of Topo I was done with a 5 min treatment with 50 ng/μl of Topetecan (40). dl-Serine Hydroxymate (SHX, Sigma CAS number 55779-32-3), an inhibitor of seryl-tRNA synthetase which triggers the stringent response and prevents new rounds of replication, was used at a 10mg/ml working concentration for 90 min. The efficiency of the drug is checked by FACS. Rifampicin was used for 10 min at a 100 ng/μl working concentration to inhibit transcription.

**Flow cytometry:** The amount of DNA was monitored using either a BD fortessa cytometer with a 488 nm argon laser and 515–545 nm emission filter (Figure 3) or a Beckman cytoflex with a 561 nm laser and a 610/20 filter (Supplementary Figure 1) at a maximum of 5000 event per second. Calibration was done with the samples of the stationary phase of the *WT, parCts* at 25°C and *parEts* at 30°C. For all samples, ∼10^8^ cells were fixed in 70% EtOH, washed, marked with propidium iodide (2–20 μg/ml), washed again, and resuspended in sterile 1× PBS pH 7.2 prior to analysis. FCSalyzer software (https://sourceforge.net/projects/fcsalyzer/) was used for data analysis.

**Hi-C libraries:** Hi-C libraries were generated as recently described in (41). 30 ml of culture was grown in Minimal Medium A supplemented with casaminoacids and glucose until OD_600 nm_ ∼0.2. Protein–DNA interactions were cross-linked by the addition of 37% formaldehyde (3% final concentration) for 30 min at room temperature with gentle agitation. Crosslinking was quenched with 2.5 M glycine (0.4 M final concentration) for 20 min at room temperature with gentle agitation. Fixed cells were then collected by centrifugation (4000 × g, 10 min 4°C), washed once in 1× PBS and snap frozen on dry ice and stored at –80°C until use. To proceed to the digestion of the cells, pellets were thawed on ice and resuspended in 1.2 ml of 1× TE+ complete protease inhibitor (EDTA-free, Roche). Cells were transferred to a VK05 tubes containing glass spreads, and then lysed with precellys (V750; 5 × 30 s). Uncross linked proteins were then solubilized by incubating the lysed cells with 10% SDS (0.5% final) for 10 min at room temperature. 1 ml of lysed cells was then added to the digestion mix (3 ml of dH_2_O, 0.5 ml of 10× NEBuffer1, 0.5 ml of 10% Triton-X 100 and 1000U HpaII) and incubated 3 h at 37°C. Digested cells were pelleted by centrifugation and resuspended in 398 μl of water before being added to the biotinylation mix (10× ligation buffer without ATP; dAGTtp 3.3 mM; biotine-14-dCtp 0.4 mM; 50U klenow (NEB5U/μl)) and incubated 45 min at 37°C with agitation. Ligation mix (10× ligation buffer without ATP; BSA 10 mg/ml; ATP 100 mM; 250 U ThermoFisher T4 DNA ligase) was then added to the biotinylated DNA and the mix was incubated 3 h at room temperature with gentle agitation. Protein–DNA complexes were then reverse crosslinked by adding the 20 μl of EDTA 0.5 M, 80 μl of SDS 10% and and 100 μl of proteinase K 20 mg/ml and incubating at 65°C overnight. DNA extraction is made by phenol–chlorophorm and precipitation with ethanol. DNA is washed with 70% ethanol, resuspended in 130 μl of TE and then treated with 20 mg/ml RNAse for 30 min at 37°C. Samples were sonicated using a Covaris S220 instrument to obtain fragments between 300 and 500 bp, then purified by AMPure XP beads. The Illumina process was performed according to manufacturer recommendation, with are 12 cycles of amplification. The size of the DNA fragment in the libraries are checked on TAE 1% agarose gel and subjected to paired-end sequencing on an Illumina sequencer (NextSeq500–550–75 cycles).

**Generation of contact maps:** Generation of contact maps was done with *E. coli* analysis pipeline developed by Axel Cournac (https://github.com/axelcournac/EColi_analysis). Briefly, reads recovered from the sequencing were aligned with bowtie2 on the reference *E. coli* genome (NC_000913), the two ends were merged and the reads were filtered to have a mapping quality strictly >30. Then each read was assigned to a HpaII restriction fragment (fragment attribution), >80% of the reads on average are conserved as informative reads. The contact matrices files are then generated, binned at 2, 5 or 10kb and normalized through the sequential component normalization procedure (SCN (42)).

**Ratio matrices and ratio plots:** In order to visualize the differences between two contact maps, their ratio is calculated as described in (12). Briefly, contacts made in the mutant map are divided by the contacts made in the control map for each loci. Increase or decrease of contact in the mutant compared to the control will appear red or blue respectively and no changes will appear white. To visualize the contact signal at a smaller scale we use the ratio plot representation, as described by (12). Briefly, the ratio-plots summarize the differences of contacts between two conditions made by each bin with its neighboring bins, regardless of their orientation. For each condition, the average contacts (AC) made by a bin along the genome with bins upstream and downstream at increasing distances (from 5 kb to 1000 kb or to 2000 kb, in 5 kb increments), was computed The log_2_(ACmutant/ACwt) was then displayed as a heatmap.

**Viability assay:** For each strains, cells were serially diluted in LB (10^-1^ to 10^-8^) and 1μl of each dilution is deposed on LB agar plates. Plates were incubated overnight at 30°C.

**DNA density analysis:** Cells were grown using the same conditions as Hi-C. 1 ml of culture of a control sample at 30°C and a sample after a 60 min shift at 42°C for each strain were fixed every 20 min (t0–t60), using an equal volume of 1× PFA (37% paraformaldehyde + glutaraldehyde + 1× PBS). DAPI (0.5 μg/ml) is added and the mixture is incubated 10 min in the dark. Cells were then washed and deposited on microscopy slides containing a freshly made agarose pas (1× PBS + 1% agarose). Samples were observed with a Spinning disk (YokogaWa) W1 system on a Zeiss inverted confocal microscope with a 63×, phase objective. To avoid bleaching, each field of view was only observed in the phase contrast channel before acquisition. Using ImageJ, the density of DAPI fluorescence in each nucleoid was calculated as a proxy for nucleoid density.

**Loci positioning analysis:** Cells were grown akin to Hi-C samples (above). For the experiments with the *parE^ts^* allele, 1ml of culture of a control at 30°C and a sample after shift at 42°C for each strain, were fixed every 20 min (t0–t60), using an equal volume of fixation medium (3.7% formaldehyde, 0.006% glutaraldehyde, 1× PBS). Cells were pelleted and resuspended in 100 μl of fresh Minimal medium A. Three drops of 2μl are deposited on glass slides containing a freshly made agarose pad (1× PBS + 1% agarose). Samples were observed with a Spinning disk (Yokogawa) W1 system mounted on a Zeiss inverted confocal microscope at 630-fold magnification. The position of foci in the cell in each condition was analyzed with the MicrobeJ (https://www.microbej.com/, (43)) and ObjectJ plugins of ImageJ https://sils.fnwi.uva.nl/bcb/objectj/. Two color localization was performed in *wt* and *parCts* strains with 7 couples of tags forms with combination of *gusC parS pMT1* (ter), *aidB parS P1* locus (ori), ygeB *parS P1 (left), yfgE parS pMT1 (left 2), yoaC parS pMT1, gusC parS P1, crl parS P1* and *cynX parS pMT1* tags (44). Cells were grown at 25°C in Minimal medium A supplemented with casaminoacids and glucose until culture reached OD = 0.1 and then cells were shifted to 30°C for 1 h. Cells were observed live on agarose pad on a thermo-controlled stage with a Zeiss inverted epifluorescence microscope equipped with led illumination. Inter focal distance distributions correspond to the average of 3 independant experiments. An average of 600 cells were analyzed per strain.

## RESULTS

### Topo IV inactivation induces large-scale chromosome conformation changes in enterobacteria

To investigate the respective contribution of each topoisomerase to genome-wide bacterial chromosome organization, we applied the capture of chromosome conformation (Hi-C) methods (41,45) (Material and Methods) to exponentially growing *E. coli* cells mutated or inactivated for each of the four topoisomerases. The ratio between *wt* and mutant/depleted cells contact maps were plotted (Materials and Methods) (12).

**Figure 1.**
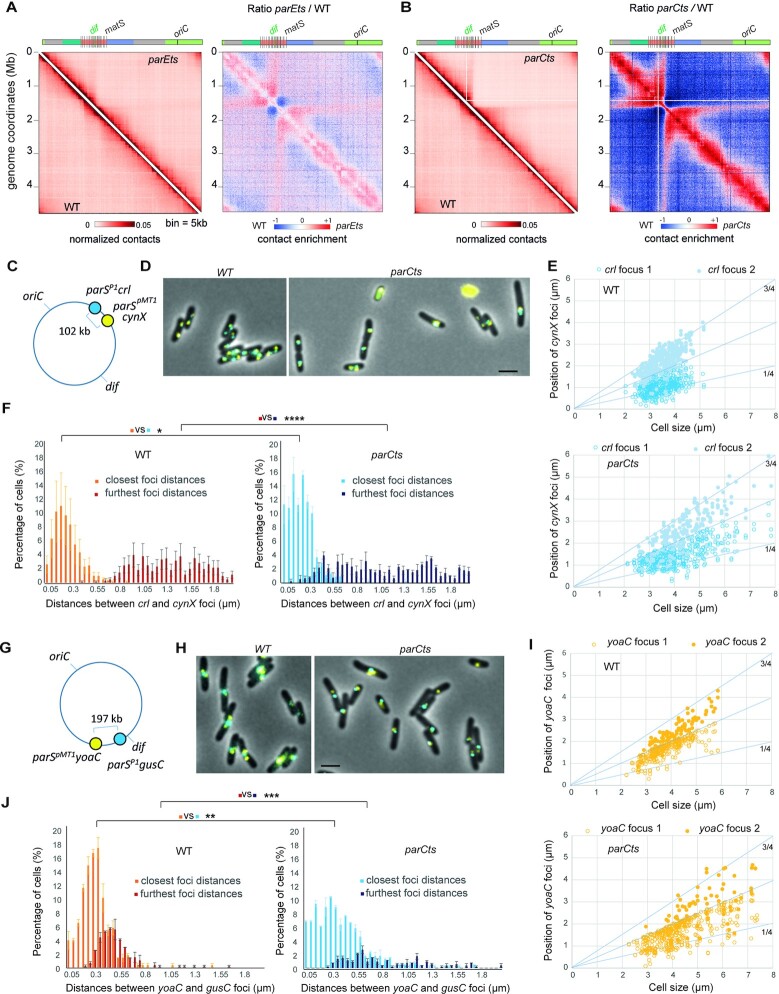
Impact of Topoisomerase IV alteration on nucleoid organization of E. coli. (**A**) Hi-C analysis of the wild type (*wt*) and *parE^ts^* strains. For each panels, symmetric halves of the normalized contact map binned at 5kb with the *wt* on the bottom and the *parE^ts^* after 60min of shift to non-permissive temperature (42°C) on the top, and the corresponding ratio matrix. (**B**) Hi-C analysis of the *parC^t^*^s^ strain after 60min of shift to non-permissive temperature (30°C). Genome coordinates are indicated by the x and y axes. Macrodomains are represented by light green (*ori*), dark green (*right*), red (*ter*), blue (*left*), gray (NR/NL). *matS* sites are represented as grey bars. For the normalized contact maps, the color scale of the frequency of contacts between two regions of the genome is indicated below (arbitrary units), from white (rare contacts) to dark red (frequent contacts). For the ratio matrices, a decrease or increase in contacts in the mutant cells compared with the control is represented with a blue or red color, respectively. White indicates no differences between the two conditions. (**C**) Schematic representation of the loci tagged with *parS*/ParB fluorescent reporters in the *right* replichore and used for the experiments presented in (D) to (F). (**D**) Representative fluorescence microscopy images of the *wt* and *parC^ts^* strains tagged with *parS*/ParB systems after 1 h at 30°C. Scale bar is 3 μm. (**E**) Localization analysis of the *crl* foci after 1 h at 30°C. (**F**) Analysis of the inter focal distances between *crl* and *cynX* foci. For the cells containing two foci of one or both tags the closest and furthest distances were plotted. *N* = 3 replicates of 200 cells each, we used an F test to compare variances of the closest and furthest distances in *wt* and *parCts* strains. (** P<0.005, *** P < 0.0005, **** P< 0.00005). (**G–J**) same legend as (C) to (F) for the *yoaC* and *gusC* loci located in the terminus region.

The inactivation of Topo IV using thermosensitive alleles of either the *parC* (*parC^ts^*) or *parE* (*parE^ts^*) subunits (33) resulted in significant changes in the global chromosomal architecture (Figure 1A and B). Chromosome folding changes were observed upon a 1h shift at 30 or 42°C respectively for the *parC^ts^* and the *parE^ts^* mutants. At these temperatures viability and DNA content were modestly affected (Supplementary Figure 1). We observed for both mutants changes in mid- to long-range DNA contacts in three regions of the genome: First, an increase in short- to medium-range contacts (50–300 kb) from *oriC* to positions located at ∼1 Mb and ∼2 Mb on the right and left replichores, respectively. Second, a strong reduction of mid-range contacts in the terminus region (coordinates 1315–1830 kb, Figure 1A, B, Supplementary Figure 2) accompanied by an increase of very short-range contacts (<50 kb, 10 bins). This particular terminus pattern also exhibits a strong (CID-like) border around the *dif* site. Finally, a peculiar butterfly-like signal with two wings of long-range contacts emerges from the flanking regions of the Ter, extending towards *oriC*. These changes in chromosome folding are temperature dependent, reproducible and rescued by a plasmid expressing ParE into the *parE*^ts^ mutant cells (Supplementary Figure 3A–C). To broaden these observations, we applied Hi-C to another gamma-proteobacterium, *Salmonella typhimurium* for which thermosensitive Topo IV mutations are available (46,47). Similar conformational changes were observed (Supplementary Figure 3D and E).

To assess the specificity of these signals, we disrupted the function of the three other topoisomerases of *E. coli*. To reduce Topo I activity, we used the *topA31* allele (48), a *topB* deletion mutant was used to study Topo III (49), and DNA gyrase was studied using a thermosensitive mutant of *gyrB* (50). In contrast to Topo IV alterations, inactivation of Topo I and gyrase activity resulted in a decrease of short-range contacts. These observations are consistent with the involvement of supercoiling in the definition of CIDs (9). Alteration in Topo III resulted in a slight gain in short-range contacts, with no other significant changes in the chromosome conformation of these cells detected (Supplementary Figure 3F–H). Altogether, our observations suggested that the genome folding changes observed in the *parE^ts^* and *parC^ts^* strains were genuine consequences of the alteration of Topo IV activities.

### Hi-C features observed in *Topo IVts* mutant cells are concomitant with nucleoid reorganization

We complemented our Hi-C approaches with fluorescence imaging of individual genomic loci. We selected *parS*/ParB systems that have been extensively validated in *E. coli*. Unfortunately, the very poor fluorescence yield of the lowly expressed ParB P1-CFP tag at 42°C did not allow us to perform experiments with the *parE^ts^* strain. We focused therefore on experiments with the *parC^ts^* strain 1 h upon shift to 30°C. Imaging further supported that nucleoid organization and compaction undergoes drastic changes following *parC^ts^* inactivation. The number and positioning of different *parS*/ParB chromosomal tags were observed (Figure 1C-J and Supplementary Figure 4). In the *WT* cells, upon shift to 30°C, the two copies of loci close to *oriC* preferentially localized around the ¼ and ¾ positions and loci located on the side of the chromosome arms located at the ¼ and 60% positions following the canonical L–R–L–R pattern. Loci close to the *terminus* localized to the new pole in newborn cells and at mid-cell in older cells. In the *parC^ts^* strain, the number of cells with two copies of each fluorescent tag decreased and the cell age at which this second copy appears was delayed (Figure 1E and I). Since replication is only modestly perturbed by Topo IV alteration in the *parC^ts^* strain at 30°C (Supplementary Figure 1B), this might reflect segregation defects. Localization of the tags was also perturbed, with most *oriC*, *left* and *right* foci localizing closer to mid-cell in between the ¼ and ¾ positions. Simultaneously *ter* foci frequently migrated away from mid-cell. These observations suggest that global chromosome conformation was affected and / or cell cycle course was perturbed.

Simultaneous visualization of two fluorescent tags allowed us to measure interfocal distances in between distant genomic loci, a proxy for genome compaction (Figure 1F and J). First, we analyzed the distances separating close loci (102 and 197 kb). In the *parC^ts^* strain at 30°C, we measured a significant shift of the distribution of the distances between the *crl* and *cynX* tags (Figure 1F) and the *yoaC* and *gusC* tags (Figure 1J) toward short distances. Since the distances of both close and far couples were reduced we postulate that it reflects both inter and intra sister chromatid compaction. These observations agree with the increased frequency of short- to medium-range contacts observed with Hi-C experiments.

### Kinetic analysis of genome folding changes imposed by crippling of Topo IV

**Figure 2.**
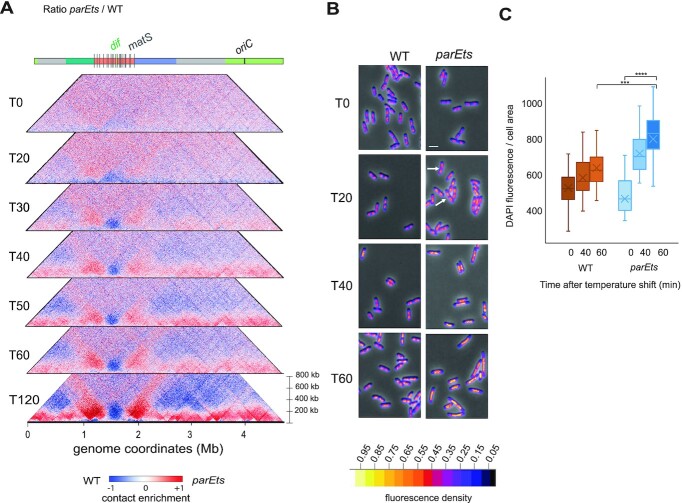
Hi-C features observed in *parE^ts^* mutant cells established progressively as nucleoid reorganized. (**A**) Kinetics of the impact of Topo IV alteration with a time point every 10 min after shift at non permissive temperature from *t*0 to *t*60 represented on the ratio matrices of *parE^ts^* cells versus *wt* cells. A decrease or increase in contacts in the mutant cells compared with the control is represented with a blue or red colour, respectively. White indicates no differences between the two conditions. (**B**) Merged images of phase contrast and DAPI signal (the density of the DNA signal is represented with a fire color scale) of *wt* (left) and *parE^ts^* (right) cells every 20 min during a temperature shift. Arrows indicate the merging of nucleoids in the *parE^ts^* cells. Scale bar = 2 μm. (**C**) Quantification of the fluorescence density as proxy for DNA density across time of the *wt* (brown boxes) and *parE^ts^* (blue boxes) (a Student's test, ***P < 0.0005).

We performed a kinetic analysis of chromosome reorganization using Hi-C and imaging, of chromosome folding after Topo IV inactivation. Hi-C revealed progressive increase in short- to mid-range contacts, which became prevalent along chromosome arms after 40 minutes (Figure 2A). Concomitantly the butterfly wings expanded from positions 1 and 2 Mb. The *terminus* region behaved differently, showing a persistent lack of short-range interactions over the entire kinetics (Figure 2A). DAPI staining revealed that the nucleoid reorganized with a comparable kinetics. In the first 20 min, we observed a global expansion of the DAPI signal leading to the apparent of fusion of the different DNA masses present in a single cell (Figure 2B). At later time-point the DNA density increased in the nucleoid and it relocalized to mid-cell leaving empty large polar regions. In a 60 min period at 42°C the average DAPI staining density increased by 1.5-fold compared to *wt* (Figure 2C). Altogether, these time course experiments show that while nucleoid progressively reorganize upon inactivation of Topo IV, specific contacts accumulate at discrete positions surrounding the *ter* domain. Therefore that Topo IV plays a role in suppressing the formation of short- to medium-range and long-range contacts within or between sister chromosomes.

### The absence of Topo IV activity results in the co-localization of distant loci with the terminus of the chromosome

**Figure 3.**
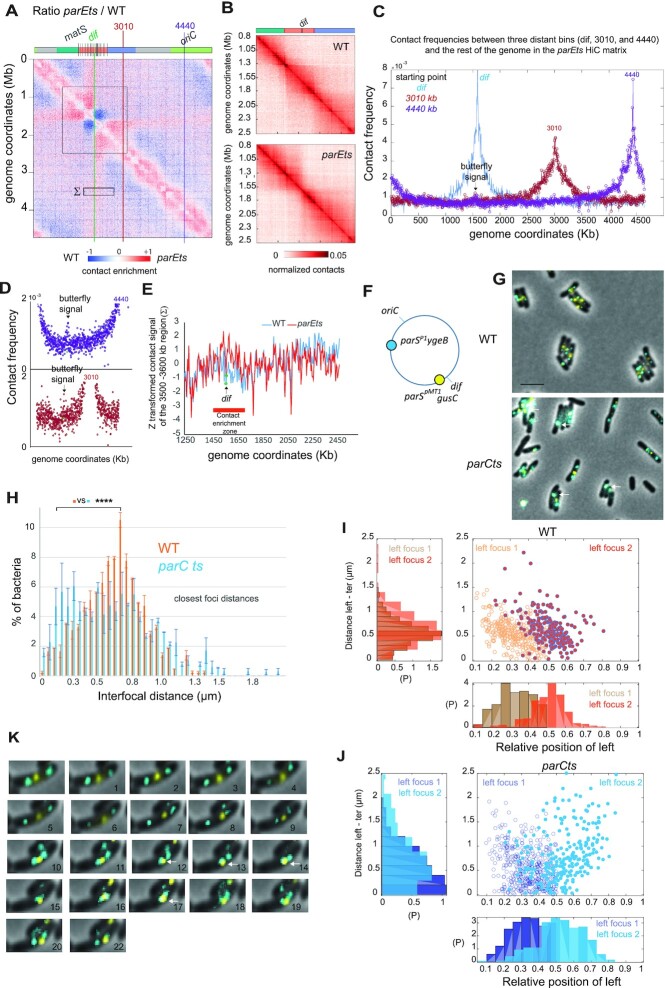
Characterization of the butterfly wings pattern. (**A**) Ratio of the normalized contact map of *parE^ts^* and *wt* where the butterfly wings borders are indicated by gray lines (1.3 Mb – *dif* and *dif* – 1.8 Mb) and two representative zones at 3301 kb (red) and 4440 kb (purple). (**B**) Zoom on the terminus macrodomain of the *wt* and *parE^ts^* matrices from 0.8 to 2.5 Mb. (**C**) Frequency plot of contacts from the *dif*, 3010 and 4440 kb positions. This representation, similar to a 4C analysis, allows direct measurement of all the contacts made by a given region with the rest of the chromosome. (**D**) Rescaling of the plot C to show the butterfly wing contacts in the dif area. (**E**) Z transformation of the contacts made by a 100 kb region located between the coordinates 3500 and 3600 kb (marked Σ in A) with the terminal part of the genome (coordinates 1250–2450 Kb). *Wt* and *parE^ts^* normalized matrix were analyzed.) (**F**) Schematic representation of the loci tagged with *parS*/ParB fluorescent reporters in the left replichore (*parS^P1^ygeB*) and the terminus region (*parS^pMT1^gusC*) used for the experiments presented in (G) to (H). (**G**) Representative fluorescence microscopy images of the *wt* and *parC^ts^* strains tagged with *parS*/ParB systems after 1 hour at 30°C. Arrows indicate colocalized *left* and *ter* foci. Scale bar is 3 μm. (**H**) Distribution of the nearest interfocal distance between *ter* and left foci in the *wt* and *parC^ts^* strains after 1 h at 30°C, we used an F test to compare variances of distances in *wt* and *parC^ts^* strains (*** P < 0.0005, N = 3 replicates of 200 cells each). (**I**) Scatter plot representing the distance between *ter* and *left* foci (cells containing two *left* foci, were oriented to plot the *left one* focus in the first half of the cell) according to the positioning of the *left* focus in *wt* cells. Density of points along the *x* and *y* axes in the scatter plot is represented on the histograms. (**J**) Same as I in the *parC^ts^*strain. (**K**) Twenty two minutes timelapse experiment illustrating transient contacts between the *left* and *ter* foci. Arrows indicate exact colocalization.

We characterized further the two butterfly-wing stripes emanating at a 45° angle from the edges of the *terminus* region in the ratio contact maps between Topo IV and *wt* (Figure 3A coordinates 1.3–1.5 Mb and 1.5–1.8 Mb, respectively). These signals correspond to an enrichment in medium and long-range contacts by ∼200 kb regions that in one direction span all the way up to *oriC*, while on the other direction, are blocked by *dif*, which represents a barrier preventing contacts across each halves of the *terminus* (Figure 3A and B). Measuring the contact frequency of individual bins with the rest of the genome, we investigated how each 5kb bin interacts with the terminus region and therefore contribute to the butterfly signal (Figure 3C and D). As illustrated for two representative regions (position 3010 and 4440 kb), a small (≈1.5-fold), but significant, contact enrichment with the *dif* zone was detected several megabases away from *dif*. This enrichment involved several (4 to^10^) bins symmetrically localized around *dif*. The contacts between the *dif* zone and the 3010 kb or 4440 kb regions present a comparable frequency, suggesting that they do not obey to genomic distance law. We observed, on zoomed panels, that the bin containing *dif* (green dots on Figure 3D) was not involved in the butterfly wing contacts (Figure 3D). To test this exclusion of *dif*, we measured the sum of contacts made by a 100 kb window 2 Mb away from *dif* (Σ sign of Figure 3A) with the 1.2 Mb region containing *dif* (Figure 3E). This window displayed enriched contacts with most bins from the *terminus* region, but not with *dif* itself. Their distance independent frequency and the lack of asymmetry at *dif* strongly suggests that butterfly wings correspond to long-range 3D contacts rather than slithering events. To confirm the propagation of these very long-range contacts at the single cell level, the localization of fluorescently labelled loci positioned in the left replichore (*ygeB* locus) and *terminus* region (*gusC* locus*)* were observed in cells carrying the *parC^ts^* allele (Figure 3F, G), that displayed butterfly wings at low temperature (30°C) (Figure 1C). In these conditions, the *left* and the *ter* loci colocalized (interfocal distance < 250 nm, arrows on Figure 3G) more frequently than for the *wt*. About 18% of the *parCts* cells show a colocalization of the *left* and *ter* foci compared to 9% in the *wt*. Similar changes in the distribution of distances between *ori* and *ter* or *right* and *ter* foci were observed (Supplementary Figure 4). Interestingly, the distances between *left 2* and *right* loci did not change; it suggests that it does not correspond to a global compaction but rather a particular genome folding involving the *terminus* (Supplementary Figure 4). Colocalized *ter* and *left* foci were observed at various positions in the cell but are slightly more frequent near mid-cell (Figure 3I and J). Time-lapse experiments showed that *left* and *ter* foci colocalization was transient and involved a rapid migration of the *left* focus toward an immobile *ter* focus (Figure 3K). As illustrated on the representative example the left*-ter* contact did not induce a duplication of the *ter* or *left* foci (Figure 3K). The rapid photobleaching of the CFP tag did not permit to investigate quantitatively these movements and to attribute them to a particular step of the cell cycle of the *parCts* strain grown at 30°C. Imaging confirms the existence of long-range contacts between the *terminus* and distant regions of the chromosome in absence of Topo IV activity. The detection of *ori, left* and *right* occasional colocalization events with the *terminus* in *wt* cells (Figure 3I and Supplementary Figure 4) suggests that the peculiar genome folding events detected in the absence of Topo IV activity might also exist in some *wt* cells. To evaluate a putative role of such contact in chromosome segregation, we measured the distance between sister *left and* sister *ori* foci as a function to their distance with the *ter* focus. In *wt* cells the shortest distances between sister *left* and sister *ori* foci were observed at mid-cell when these foci were the closest from the *ter* focus (*R*^2^ = 0.18 and 0.16, respectively for *left* and *ori*) (Supplementary Figure 5). Although more *left* and *ori* foci colocalized with the *terminus* in the *parC^ts^* strain, the localization pattern of newly duplicated sister *ori* and sister *left* foci was significantly perturbed (*R*^2^ = 0.08 and 0.006). These observations suggest that duplication of genomic loci might be synchronized or triggered by a ‘contact’ with the *terminus* near mid-cell; the butterfly wings pattern (Figure 3A–D) and the associated extra colocalization with the *terminus* (Figure 3G and H) may therefore correspond to an accumulation of unsuccessful duplication attempts.

### *parE^ts^* inactivation increases inter-chromosomal contacts

**Figure 4.**
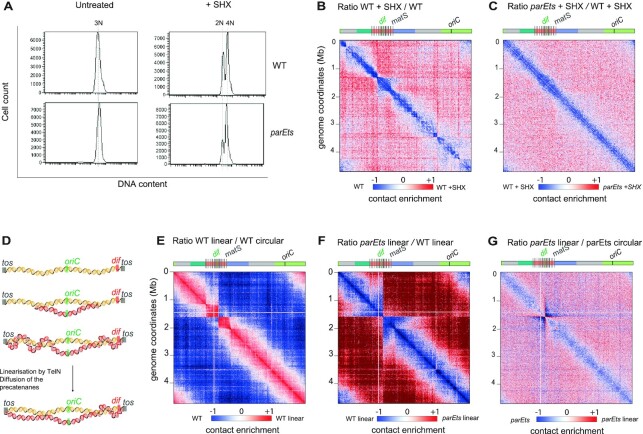
Hi-C features resulting from *parE^ts^* alteration result from sister chromosome interactions. (**A**) Flow cytometry assay of *wt* and *parE^ts^* cells after 60 min of shift at 42°C untreated (left) and treated with 10 mg/ml of SHX for 90min total (right). Nucleoid were stained with propidium iodide. Ratio of normalized contacts map binned at 5 kb of (**B**) Hi-C ratio matix of the *wt* + SHX (non-replicating cells) compared to *wt* (replicating cells), (**C**) Hi-C ratio matix of the *parE^ts^* + SHX (non-replicating cells) compared to *wt* + SHX (non-replicating cells). (**D**) Graphic representation of the replication in *E. coli* linear strain. The chromosome is linearized via the tos / TelN system of the phage N15. *tos* sites (gray box) were inserted in the terminus macrodomain. The replication starts at the oriC site (green box) and progress in a bidirectional manner toward the dif site (red box). We hypothesis that precatenanes are formed during the replication of the linear chromosome in the same manner as in the circular one. Sister chromatids are represented by double helices in red (sister A) and yellow (sister B). Before chromosome segregation, the protelomerase TelN linearize the sister chromosomes at the tos site which could possibly allow for diffusion and resolution of the majority of the precatenanes. Ratio of normalized contacts map binned at 5 kb of (**E**) linear *wt* vs circular *WT*, (**F**) linear *parE^ts^* vs linear *wt* and (**G**) linear *parE^ts^* linear versus *parE^ts^* circular. Macrodomains and interesting positions of the genome are indicated above the plot akin to Figure 1. The y axis indicates the genomic coordinates. A decrease or increase in contacts in the mutant cells compared with the control is represented with a blue or red colour, respectively. White indicates no differences between the two conditions.

The ability of conventional Hi-C to capture intra-chromosomal (*cis*) contacts is well established. However, its ability to reveal *trans* contacts between sister-chromatids remains limited given the difficulty to distinguish both homologous molecules without incorporation of modified bases (51). Since Topo IV is mainly known for its involvement in the removal of inter-chromosomal links (or catenanes), presumably between allelic (or near-allelic) loci, we sought to test whether the emergence of prominent Hi-C signals spanning throughout the *parE*^ts^ contact map could result from *trans* contacts. We therefore sought to distinguish the contribution of intra- versus inter-molecular contacts to these patterns by determining the impact of DNA replication in absence of Topo IV activity. To do this, we blocked replication initiation by treating *wt* and *parE^ts^* cells with the amino acid analog dl-serine hydroxamate (52). Upon SHX addition the distribution of DNA in *wt* and *parE^ts^* cells shifted from a single normal distribution centered around 3N genomes to two peaks respectively centered at 2N and 4N, this suggests that SHX blocked replication initiation but allowed ongoing replication rounds to finish (Figure 4A). Following SHX treatment, non-replicating cells were then shifted to 42°C for 1 h. Hi-C ratio maps of replicating versus non-replicating *wt* cells revealed a significant decrease in mid-range contacts along the entire chromosome in the absence of replication (Figure 4B). Non-replicating *parE*^ts^ cells did not show increasing mid-range contacts along the diagonal and did not show the butterfly wings pattern observed for replicating cells (Figure 4C and Supplementary Figure 6A and B).

Since the stringent response mediated by SHX also reduces transcription of many genes (53), we tested whether absence of the characteristic pattern in SHX-treated cells could result from a poor transcription. Asynchronously growing *parE*^ts^ and *wt* cells were therefore treated with the antibiotic rifampicin to inhibit transcription. The partition of the chromosome into three regions in *parE*^ts^ cells remained visible upon inactivation of Topo IV at 42°C (i.e. the butterfly wings, a higher mid-range contacts in the *oriC* proximal region, and lower mid-range contacts in the *ter*). However, their intensity was reduced. At this stage, it is not possible to distinguish between specific effects of transcription on the pattern mediated by Topo IV alteration or a more trivial alteration of Hi-C matrices’ quality by rifampicin (9), (Supplementary Figure 6C, D). Taken together, these results suggest that Topo IV inactivation induces characteristic Hi-C patterns in replicating and transcribing cells, presumably because they correspond to inter-chromosome contacts.

### Butterfly wings signals are linked to chromosome topology

We then wondered whether the circular nature of the *E. coli* chromosome could lead to topological constraints on the *terminus* region that would translate into specific contact patterns in the absence of Topo IV. To test this, we performed Hi-C experiments in strains carrying a genome linearized at a *tos* site, inserted near *dif*, thanks to the bacteriophage N15 telomerase (54). In this strain, Topo IV is still required for growth (54). However, it is no longer active at *dif*, and the filamentation phenotype of the *matP* mutant is now rescued (37). These observations hinted that topological tension resulting from Topo IV inactivation freely diffuses when the N15 telomerase linearizes the duplicated chromosome (Figure 4D). In the *wt* strain, linearization of the chromosome near *dif* resulted in a notable redistribution of long-range contacts towards short and mid-range contacts (Figure 4E and Supplementary Figure 6E). Upon inactivation of *parE^ts^*, the Hi-C contact maps of the linear chromosome displayed a significant loss of short- to mid-range contacts, no butterfly wings and no *terminus* pattern compared to circular *wt* chromosomes (Figure 4F and Supplementary Figure 6F). The comparison between *parE^ts^* strains carrying either a linear or a circular genome further revealed a decrease in short/medium contacts along linear chromosomes, in sharp contrast to *wt* conditions, and compatible with a reduction in the number or density of precatenation links (Figure 4G). This suggests that the linearization of the strain suppresses the accumulation of interminglements between the sister chromatids. Altogether, these observations strongly support the hypothesis that the enhanced mid-range contacts along chromosome arms, as well as the butterfly wings signals, form in response to a failure of Topo IV to remove inter-chromosomal links.

### Topo III activity partially rescues Topo IV deficiencies on chromosome conformation

**Figure 5.**
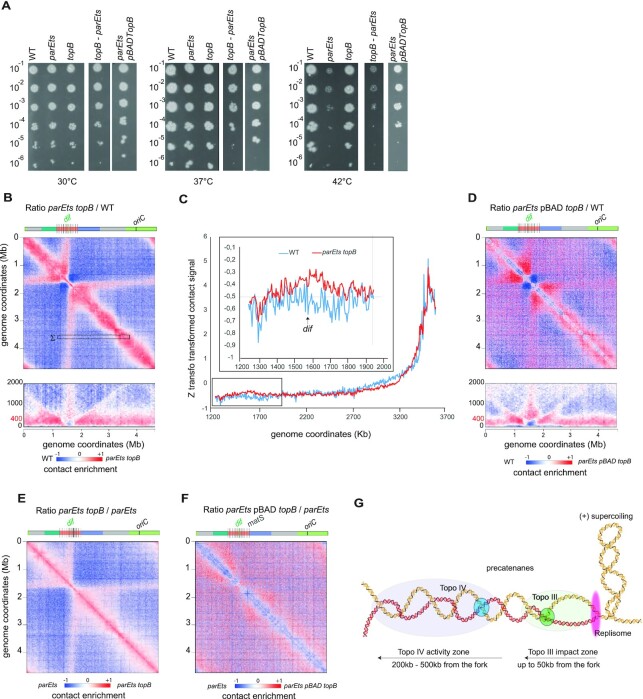
Topo III activity partially rescues Topo IV deficiencies on chromosome conformation. (**A**) Viability assay performed by droplet CFU of the *wt*, *parE^ts^, parE^ts^ topB, topB*, and *parE^ts^* pBAD *topB* at 30, 37 and 42°C. (**B**) Ratio of normalized contacts map binned at 5 kb of for the *parE^ts^ topB* vs *wt* matrix. Macrodomains and interesting positions of the genome are indicated above the plot. The y axis indicates the genomic coordinates. A decrease or increase in contacts in the mutant cells compared with the control is represented with a blue or red colour, respectively. White indicates no differences between the two conditions. (**C**) Z transformation of the contacts made by a 100 kb region located between the coordinates 3500 and 3600 kb (marked Σ in B) with the left replichore (coordinates 1250–3700 kb). *wt* and *parE^ts^ topB* normalized matrix were analyzed. Inset, zoom on the terminal part of the genome. (**D**) Ratio of normalized contacts map binned at 5 kb of for the *parE^ts^ topB* versus *parE^ts^*. (**E**) *parE^ts^* pBAD *topB* versus *wt*. (**F**) *parE^ts^* pBAD *topB* versus *parE^ts^*. (**G**) Graphic representation of the decatenation activity zone of Topo IV (blue) and Topo III (green) after the replication fork (pink). Sister chromatids are represented by double helices in red (sister A) and yellow (sister B). Topo III is able to act directly behind the replication fork and only impacts contact in a 50 kb zone. Precatenanes beyond the 50 kb zone, are dealt with by Topo IV which impact contacts 200–500 kb away from the replication fork.

Topo III (*topB*) is a type I topoisomerase whose role during the normal cell cycle remains unclear. Both the modest chromosome conformation changes (Supplementary Figure 3G) and normal viability observed in the mutant (Figure 5A) confirm that in laboratory growth conditions its role is limited. However, part of its activity is revealed when Topo IV is impaired, as Topo III overexpression rescues *parE^ts^* growth defects (Lee *et al.*, 2019; Nurse *et al.*, 2003; Figure 5A), whereas *topB* deletion reduces the viability of *parE^ts^* strains at intermediary temperatures (Figure 5A). When *parE^ts^* was inactivated in a *topB* deletion strain, the Hi-C pattern observed in *parE*^ts^ mutant cells at the non-permissive temperature was strengthened (Figure 5B), with mid-range contacts increase, and stronger butterfly and *terminus* patterns (Figure 5B). In the *topB parE^ts^* strain, long-range contacts were observed between distant regions and a large central region of the ter macrodomain (Figure 5C). Their frequency was higher (Figure 5C) compared to the single *parE^ts^* strain (Figure 3C). As observed for the *parE^ts^* strain the *dif* bin appeared to make less long-range contacts than its neighbors (Figure 5C). Interestingly, *parE^ts^* features were visible in the contact maps at 30°C in the absence of Topo III (Supplementary Figure 7A), suggesting that at this temperature Topo IV is already partially inactivated, but that the organization defects are suppressed by Topo III. This is in good agreement with the CFU data (Figure 5A). Ratio plot and 4C-like analysis also revealed important changes in the distribution of short and mid-range contacts outside of the *terminus* region when both Topo IV and Topo III are inactivated. When plotting the ratio map between the *parE^ts^ topB* matrix and *topB* or *parE^ts^* matrices we characterized the contribution of Topo III and Topo IV. Butterfly wings, and mid-range contacts appeared in the *parE^ts^ topB* compared to *topB* matrices (Supplementary Figure 7B–D). On the other hand, comparing the *parE^ts^ topB* matrix with *parE^ts^* highlighted mostly an increase in short-range contacts (Figure 5D). The overexpression of Topo III rescued the viability of the *parE^ts^* strain at non-permissive temperatures (Nurse *et al.*, 2003, Figure 5A). The Hi-C contact map of a *parE^ts^* strain overexpressing Topo III at non- permissive temperature displayed a reduction in short-range contacts along the diagonal, but little changes regarding butterfly wings and mid-range contacts (Figure 5E and F and Supplementary Figure 7C and 7D). These observations demonstrate that short-range contacts are mediated by a topological structure that can be removed by Topo III, most likely precatenanes with single strand regions near the replication fork. Since butterfly wings, mid-range contact and *terminus* insulation are not suppressed by Topo III overexpression, they appear as a direct consequences of Topo IV inactivation and might therefore reflect the catenation of fully replicated molecules, a substrate impossible to unlink for Topo III (Figure 5G).

### The position of butterfly wings is determined by MatP/*matS*, the Ter macrodomain organizer.

**Figure 6.**
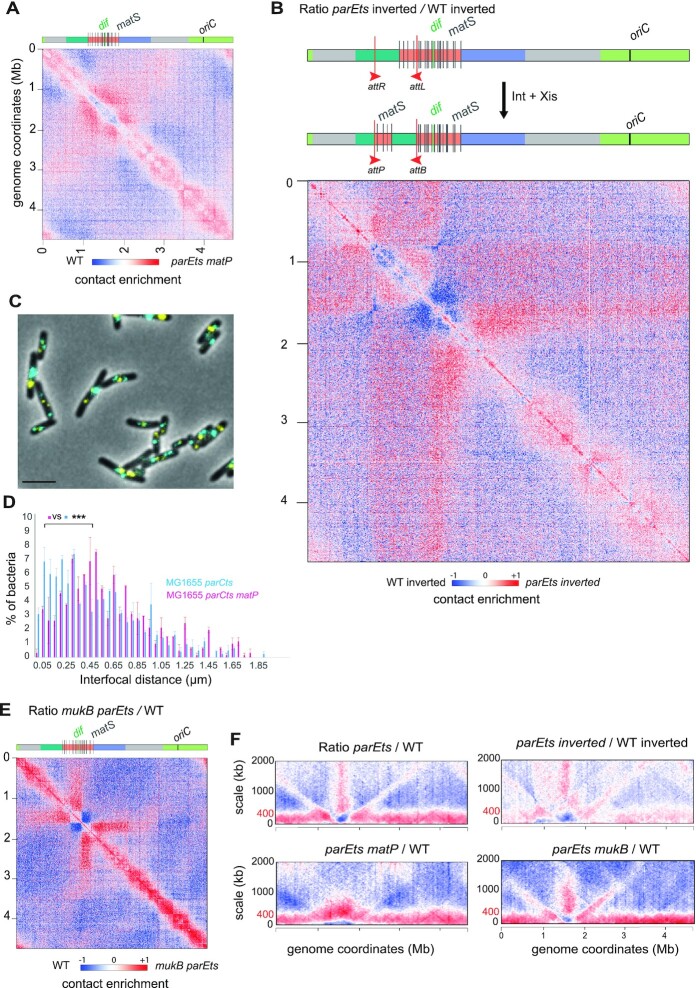
The butterfly position is determined by MatP/*matS*, the terminus macrodomain organizer and the bacterial SMC MukB. Ratio of normalized contacts map binned at 5 kb, of (**A**) *parE^ts^ matP* versus *wt*, (**B**) *parE^ts^* inverted versus *wt* inverted. The inversion was performed via the *attR-attL* system. It inverted the region between 0.8 and 1.30 Mb resulting in the displacement of four *matS* sites. (**C**) Representative fluorescence microscopy images of the *parC^ts^ matP* strain tagged in the left replichore (*parS^P1^ygeB*) and the terminus region (*parS^pMT1^gusC*) as on Figure 3F and G. Scale bar is 3 μm. (**D**) Distribution of the nearest interfocal distance between *ter* and left foci in the *parC^ts^* and *parC^ts^ matP* strains after 1 h at 30°C. We used an *F* test to compare variances of distances, *** P < 0.0005, N = 3 ). (**E**) Ratio of normalized contacts map binned at 5 kb, of *parE^ts^ mukB* versus *wt*. (**F**) Scalogram ratio of normalized contact map comparing two conditions for each 10-kb bin along the chromosome: *parE^ts^* versus *wt*, (F) *parE^ts^* inverted vs *wt* inverted as in (B), *parE^ts^ matP* versus *wt*, *parE^ts^ mukB* vs *wt*. The y axis indicates the contact distances in kB. A decrease or increase in contacts in the mutant cells compared with the control is represented with a blue or red colour, respectively. White indicates no differences between the two conditions.

The peculiar positioning of the butterfly wings at the edges of the Ter macrodomain (Figure 3A) prompted us to assess the influence of MatP on these structures. In the absence of MatP and at *parE^ts^* non-permissive temperature, the butterfly wings were replaced by a large region displaying increased long-range contacts (up to Mb distances), covering the entire *terminus* macrodomain (Figure 6A). The ratio plot of *matP parE^ts^* and *matP* contact maps further show an enrichment in mid-range contacts within the *terminus* macrodomain in absence of MatP (Supplementary Figure 8A–D). This observation confirms that the features revealed by Topo IV inactivation are structurally connected, with MatP being responsible for the positioning of the discrete boundaries limiting the entry of putative precatenation links into the *terminus* macrodomain. These borders define the characteristic butterfly structure and favors the emergence of the *terminu*s pattern.

Based on these observations, we hypothesized that displacing the *terminus* macrodomain should reorganize the Topo IV-dependent structural features. We used bacteriophage λ site specific recombination to generate an inversion that moved the region associated with the base of the right butterfly wing (coordinate: 1. 342 702 Mb) to a region now 540 kb upstream the right replichore (coordinate 0. 806 549 Mb) (55) (Figure 6B, Supplementary Figure 8E and F). This inversion resulted in the repositioning of five *matS* sites (*matS 1–4*) to a region upstream within the right replichore, which in turn resulted in the generation of a large chromosomal region (coordinates 0.8–1.3 Mb) devoid of *matS* sites and flanked by two *matS* regions. We observed a shift in the position of the right butterfly wing that coincided with the new position of the inverted *matS* sites. The butterfly wing was less precise, and long-range contacts appeared to extend from *matS*5 to *dif*. The positioning and strength of the second butterfly signal was not affected by the inversion, suggesting that the two butterfly wings are not functionally interlinked (Figure 6B, Supplementary Figure 8E and F). Imaging experiments performed in the *parC^ts^ matP* strain showed a loss of colocalization between the *left* and *ter* fluorescent tags (Figure 6C and D) compared to the single *parC^ts^* (Figure 3G and H). Although the *left* and *ter* localization remained affected by Topo IV alteration in the *parC^ts^ matP* strain (Supplementary Figure 9), the frequency of *left* – *ter* colocalization was comparable to that of the *wt* strain (compare Figures 3H and 6C). These observations suggest that the butterfly wings Hi-C signal corresponds to genome reorganizations involving the *terminus* and orchestrated by MatP binding to *mat*S.

### MukB controls the shape of the butterfly wings

The presence of MatP in the *terminus* macrodomain inhibits MukB activity in *wt* cells (Nolivos *et al.*, 2016). In the absence of MatP, MukB is able to access the *terminus* region and has been shown to both accelerate the segregation of *terminus* loci (56) and change *terminus* conformation (12). MukB also interacts with Topo IV and modulate its activity (26,27). We therefore tested whether MukB affects the patterns observed when Topo IV is inactive. A *mukB* deletion in itself did not create butterfly pattern (12), suggesting that disrupting the ParC-MukB interaction does not alter Topo IV activity in the same way as *parE* or *parC* inactivation does. When combined with *parE^ts^*, the *mukB* mutation did not abolish the formation of butterfly wings (Figure 6E). However, their characteristics in the double mutant differ from those in the single *parE^ts^* mutant. Comparison of the *parE^ts^ mukB* mutant with the single *parE^ts^* mutant revealed that the basal portion of the butterfly wings were reinforced in the absence of *mukB* (i.e. higher frequency of contacts) and their length is reduced (Supplementary Figure 8G). In addition, we observed an increase of contacts between all regions within the butterfly, so that they now resemble a self-interacting domain (Supplementary Figure 8G), rather than a stripe (Figure 6E). Scalograms, i.e. the aggregation of contacts over various scales from each bin (12), clearly show the variations in wing shapes and sizes in the mutant strains (*parE^ts^, parE^ts^ inverted, parE^ts^ mukB* and *parE^ts^ matP*) (Figure 6F). In the *parE^ts^* strain, butterfly wings extend from the *terminus* to the origin of the chromosome. In the absence of MatP or in the inverted strain, they nearly disappear. In addition, MatP prevents the formation of mid-range contacts in the *terminus* region (Figure 6F). Finally, in the absence of MukB the butterfly wings abruptly stop ∼1 Mb from *dif* on both replichores. The absence of MukB resulted in the exclusion of the *oriC* region from the butterfly structure. Results obtained in the *parE^ts^ mukB* mutant, however, should be taken with precautions since the DNA content and replication cycle of this strain were significantly perturbed (Supplementary Figure 8H). Moreover, the poor viability of the *parCts mukB* mutant did not allow us to complement Hi-C observations with fluorescent microscopy.

The increase in short range contacts detected along chromosome arms when Topo IV is inactivated (presumably corresponding to the accumulation of precatenanes) did not change in the absence of *matP* or *mukB* (Figure 6F). This suggests that Topo IV partners do not significantly impact precatenanes dynamics. Therefore, MatP is involved in the positioning of the butterfly pattern, while MukB might determine the wing length and contact density (Figure 6F). Moreover, MatP itself and/or the resulting MukB relocalization might prevents the progression of precatenanes into the *terminus* (Figure 6F).

## DISCUSSION

### Alteration of each Topoisomerase induces specific chromosome conformations

Although DNA topology plays an important role in bacterial chromosome folding and compaction, the consequence of topoisomerase alteration on these processes are not yet fully understood. We took the advantage of Hi-C protocol improvements in bacteria to analyze chromosome conformation upon alteration of each of the four Topoisomerase of *E. coli*. Inhibition of Gyrase activity results in a strong reduction of short-range contacts over the entire genome. This agrees with previous report (9) and confirms that supercoiling homeostasis is an important driver of CID organization in bacterial genomes. Surprisingly, because they have opposite catalytic activities, Topo I inhibition also reduces short-range contacts. Topo III and Topo IV inhibitions have the opposite effects, as they both increase short-range contacts: lightly and homogeneously for Topo III, but much more significantly and with a robust patterning for Topo IV. We investigated in details the nature and determinants of this patterning.

### New chromosome contacts emerging from Topo IV inhibition are intermolecular contacts

**Figure 7.**
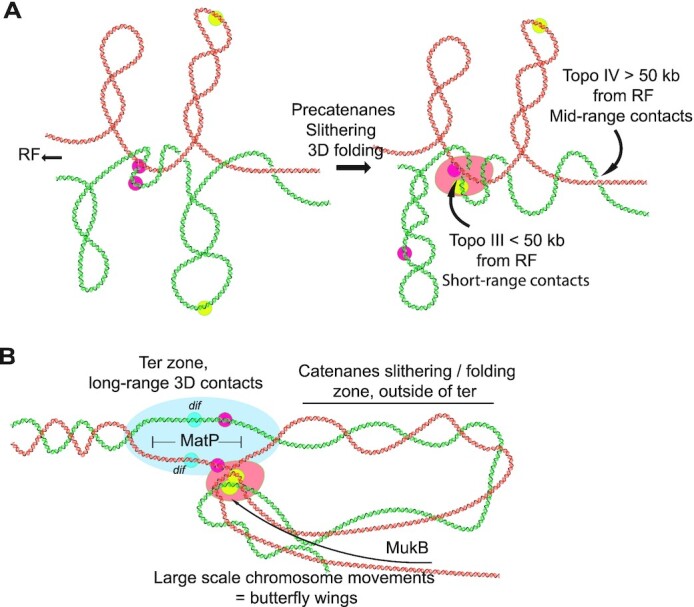
Topo IV alteration reveals that sister chromatid management involves long range genome reorganisations. (**A**) Graphic representation of putative sister chromatid precatenanes organization, dynamics and homeostasis based on Hi-C data of Topo IV and Topo III mutants. Two pairs of sister loci were represented in pink and yellow respectively. RF stands for replication forks. (**B**) Graphic representation of the terminus region and the chromosome folding events that take place around it when decatenation is impaired. This region may function as a hub where unresolved entanglements of the two sisters formed during chromosome replication enter by a yet unknown mechanism to be separated by Topo IV. The low decatenation capacity in the *parE^ts^* and *parC^ts^* strains increase the persistence/recurrence of these events and their detection with Hi-C and imaging. MukB defines the maximum distance of loci able to contact the decatenation hub.

Topo IV inhibition induces an increase of chromosome contacts in the 50–200 kb range chromosome arms with the exception of a large *terminus* region that behaves differently. We observed that these new contacts are only established in replicating cells carrying circular chromosomes. Moreover, the activity of Topo III, which appears at very short range (0–50 kb), limits their amount. Altogether, these results demonstrate that short – mid range contacts detected when Topo IV is inactivated are produced by precatenanes (Figure 7A). Their amplitude, 200–300 kb, can only be explained if catenated sister chromatids slides or fold in 3D on top of each other on a 200-300 kb window. By contrast, Topo III only modulated short scale contacts, which agrees with the idea that soon after replication sister loci are well aligned (no more than 50 kb offset). In the absence of Topo IV some of these precatenane links persist away from the replication fork, out of reach of Topo III action even when overexpressed. Therefore, a few tens of second after their replication, catenane links cannot reach the Topo III activity zone, and might be entrapped by topological barriers (Figure 7A). The comparison of wild-type replicating and non-replicating cells Hi-C matrices (Figure 4B) revealed an increase of mid-range contacts when cells replicates. This suggests that during a regular cell cycle Topo IV and Topo III sporadically let few catenation links all over the genome that will contribute to sister chromatid cohesion (32,34). Future Hi-C and sisterC (51) experiments performed on cells with synchronized cell cycle should give important insights on the nature of these inter-sister contacts and their molecular determinants.

### Very long-range contacts emerged as a butterfly pattern at the *terminus*

Butterfly wings observed in the absence of Topo IV involve contact between the *dif* area (200 kb surrounding *dif*) and the rest of the chromosome. In *wt* cells the *dif* site, in the *terminus* region, is the major Topo IV activity site, this suggests that a large number of catenation links can be removed at this locus (37,38); perhaps because they accumulate in the replication fork trap or because Topo IV is targeted there by partners such as XerCD, FtsK or MukB (57–59). It is tempting to speculate that butterfly wings’ signal may reflect a malfunctioning of a decatenation machinery active around the *dif* site in *wt* cells. The butterfly pattern is an atypical Hi-C feature; these contacts do not follow genomic distance law and are not impacted by cis barriers (Figure 3). The density of contacts inside butterfly wings is dependent on the topological status of the cell (Topo III effect) and on MukB and their position determined by MatP/*matS* (Figure 6). The butterfly wings signal correlates with an extra colocalization between the *terminus* region and distant loci in the *ori, left* or *right* regions; 10–23% in *parC^ts^* and 4–14% in *wt* cells (Figure 3, supplementary Figure 4). The colocalization events are transient (Figure 3K) and dependent on the presence of MatP (Figure 6). Even if *ori* or *left* foci colocalization events with the *terminus* were rare in *wt* cells, we observed that closest sister *ori* and *left* foci are localized at mid-cell and in the proximity of a *terminus* focus, suggesting that in some cell cycle duplication might be correlated with a contact with the terminus (Supplementary Figure 5). In this model, the *terminus* region may provide Topo IV to remove persistent catenation links associated to distant regions of the chromosome. One possible explanation for the butterfly wings’ signal could be that because Topo IV is inefficient, contacts with the terminus fail to provide decatenation and are therefore prolonged (Figure 7B). Supporting this model, we observed that butterfly wings were more robust when Topo III was inhibited (i.e. when more catenanes link sister chromatids) and less robust when Topo III was overexpressed.

Several mechanisms might drive butterfly wings’ contacts. One possibility is that a protein marking catenation links is involved in protein-protein contacts between the *terminus* and other regions of the chromosome. A DNA binding protein marking positive supercoils, GapR, has been identified in *C. crescentus* (60). A yet unknown equivalent of GapR for catenation links (catenation crossing are topologically similar to positive supercoils) might exist in *E. coli*. Future experiments will be require to test if YejK which interacts with Topo IV and binds DNA (61) or another catenane binding factor, could play such a role. Another possible explanation is that regions of the chromosome presenting excess of catenation links are excluded from the bulk of the genome. Since the absence of MukB changes butterfly wing density, one hypothesis is that catenane links cannot be extruded as plectonemic loops. As MukB does not bind to the terminus neither (25), both catenation links and terminus may be spontaneously excluded from the chromosome bulk covered by MukB and merge in a particular territory of the nucleoid.

### Chromosome Conformation Capture proposes a new read out of topological constraints

Most of our knowledge on the control of topological homeostasis by topoisomerases comes from plasmid DNA topology analyses by 1D or 2D gel electrophoresis or electron or AFM microscopy (62). Recently, molecular genomics methods were implemented to map topoisomerase activities or binding on chromosome (63). Hi-C is a popular method to study chromosome conformation, and a derivative to track inter sister contacts was recently designed in eukaryotes (51). The use of *loxP* recombination to reveal proximity of sisters (34) or their catenation (64) is also a powerful tool to decipher local chromosome topology, with a genome-wide *loxP* recombination based assay recently adapted to survey sister proximity in *V. cholera* (65). Our results suggest that Hi-C can also offer a read-out of topological constraints. Coupling all these approaches together, along with imaging, will refine our understanding of chromosome conformation dynamics during the bacteria cell cycle and particularly the contribution of inter-sister contacts.

## DATA AVAILABILITY

Sample description and raw sequences are accessible on SRA database through the following accession number: PRJNA730396.

## Supplementary Material

gkac105_Supplemental_Files

## Notes

Present address: Ingrid Billault-Chaumartin, Department of Fundamental Microbiology, University of Lausanne Quartier UNIL-Sorge, Biophore Building, CH-1015 Lausanne, Switzerland.

Present address: Hafez El Sayyed, University of Oxford, Biological Physics Research Unit, Clarendon Laboratory, Department of Physics, Oxford, OX1 3PU, UK.
