# Supplementary material for: Extended sister-chromosome catenation leads to massive reorganization of the E. coli genome
Source: Nucleic Acids Res. 2022 Feb 25;50(5):2635–50. doi: 10.1093/nar/gkac105 (PMC8934667; doi:10.1093/nar/gkac105)
Supplement: gkac105_Supplemental_Files [file gkac105_supplemental_files.zip › Supplementary_OE_rev_v1.pdf]

Genomic contacts reveal the control of sister chromosome decatenation in *E. coli*

Legend of the Supplementary figures

Supplementary Figure 1

(A) Viability in drop assay, of the WT, *parE*^{ts}, and *parC*^{ts} at 30°C, 37°C and 42°C (B) Flow cytometry of the DNA and origin contents of the WT, *parE*^{ts}, and *parC*^{ts} strains at the indicated temperatures. Replication run out experiments were carried on for 2 hours in the presence of rifampicin and cephalexin as described in (1). (C) ori:ter ratio performed by qPCR on the *wt* and *parE*^{ts} strains as described in (2)

Supplementary Figure 2

Contact frequency measurements (P(s)) according to genomic distance for the *wt, parE*^{ts}*, parC*^{ts}*, parE*^{ts} *topB* strains. Left column for the whole genome. Right colum for the terminus region (coordinates 1.3 **to 1.8 Mb).**

Supplementary Figure 3

(A) Normalized contact map of *parE*^{ts} at permissive temperature (30°C) and the corresponding ratio of *parE*^{ts} vs WT at 30°C. (B) Replicate Hi-C analysis of the wild type (*wt*) and *parE*^{ts} strains. For each experiments the ratio matrix of *parE*^{ts} vs WT 1h at 42°C was plotted. (C) Viability in drop assay, of the WT, *parE*^{ts}, and *parE*^{ts} pBR parE at 42°C. The expression of a functional ParE subunits restored the viability of the *parE*^{ts} mutant at 42°C. Expression of ParE restored the *wt* genome conformation as shown by the ratio matrix of *parE*^{ts} complemented with pBR *parE* vs WT at non permissive temperature (42°C). (D) Hi-C analysis of Topo IV aleration in *S. typhymurium.* Symmetric halves of the normalized contact map binned at 5kb with the wild type (WT) on the bottom and the *parE*^{ts} after 60min of shift to non-permissive temperature (42°C) in *S. typhymurium* on top; right panel *parE*^{ts} *S.T.* vs *wt* ratio matrix. (E) *S. typhymurium parC*^{ts} after 60min of shift to non-permissive temperature (42°C); right panel *parC*^{ts} *S.T.* vs *wt* ratio matrix. (F-H) For each panels, symmetric halves of the normalized contact map binned at 5kb with the wild type (WT) on the bottom and the altered topoisomerase on the top, and the corresponding ratio matrix. (F) Topo I, inhibited by 5min treatement with Topotecan (G) *topB* deletion and, (H) *gyrBts* after 20min of shift to non-permissive temperature (42°C). Genome coordinates are indicated by the x and y axes. Interesting positions of the genome are indicated above the plot. *matS* sites are represented as gray bars. Macrodomain are represented by light green (ori),

dark green (right), red (ter), blue (left), gray (NR/NL). For the normalized contact maps, the color scale of the frequency of contacts between two regions of the genome is indicated below (arbitrary units), from white (rare contacts) to dark red (frequent contacts). For the ratio matrices, a decrease or increase in contacts in the mutant cells compared with the control is represented with a blue or red color, respectively. White indicates no differences between the two conditions.

Supplementary Figure 4

Intracellular localization of *parS*/ParB tags in wt and parCts strains after 1h at 30°C. Each strain was tagged with a couple of *parS*/parB tags, *parS*/ParB$^{P1\text{-}CFP}$ and parS/parB$^{pMT1\text{-}yGFP}$, their positioning is shown on the corresponding cartoon. Scatter plot of the positioning of the foci along the length of the cell according to cell size. Left panel the two tags are represented, the middle and right panels respectively show the ParB$^{P1\text{-}CFP}$ and parB$^{pMT1\text{-}yGFP}$ foci. The blue line marks mid-cell. Histograms show the distribution of distances between closest ParB$^{P1\text{-}CFP}$ and parB$^{pMT1\text{-}yGFP}$ foci in both strains.

Supplementary Figure 5

(A) Intracellular positioning of sister *left parS*/ParB$^{P1\text{-}CFP}$ foci as a function of their distance to the closest ter *parS*/ParB$^{pMT1\text{-}yGFP}$ focus (top) and their relative positioning in the cell (bottom). (B) same legend as in A for the ori *parS*/ParB$^{P1\text{-}CFP}$ foci. *Wt* and *par*^{Cts} strains were analysed 1h after shift to 30°C

Supplementary Figure 6

(A) Normalized contact map binned at 5kb of WT treated with 10mg/mL of SHX for 90min at 42°C, (B) Normalized contact map binned at 5kb of *parE*^{ts} treated with 10mg/mL of SHX for 90min at 42°C, (C) Normalized contact map binned at 10kb of WT treated with 100ng/µl of rifampicin for 10min, 50h after shift at 42°C, and the ratio of WT + rif compared to WT untreated, (D) Normalized contact map binned at 10kb of *parE*^{ts} treated with 100ng/µl of rifampicin for 10min, 50h after shift at 42°C, and the ratio of WT + rif compared to WT untreated, (E) Normalized contact map binned at 5kb of *E. coli* with a linear chromosome 1h after shift at 42°C, (F) Normalized contact map binned at 5kb of *parE*^{ts} in *E. coli* with a linear chromosome 1h after shift at 42°C. Genome coordinates are indicated by the x and y axes. Interesting positions of the genome are indicated above the plot. *matS* sites are represented as gray bars. Macrodomain are represented by light green (ori), dark green (right), red (ter), blue (left), gray (NR/NL). For the normalized contact maps, the color scale of the frequency of contacts between two regions of the genome is indicated below (arbitrary units), from white (rare contacts) to dark red

(frequent contacts). For the ratio matrices, a decrease or increase in contacts in the mutant cells compared with the control is represented with a blue or red color, respectively. White indicates no differences between the two conditions.

Supplementary Figure 7

(A) Normalized contact map binned at 5kb of *parEts topB* grown at 30°C and the ratio of the mutant compared to the WT. (B) Normalized contact map binned at 5kb of *parEts topB* 1h after shift at 42°C and the ratio of the *parE^{ts} topB* compared to *topB* (normalized contact map of this mutant in Supp Fig 1I). (C) Normalized contact map binned at 5kb of pBAD *topB* grown at 30°C and the ratio of the pBAD *topB* compared to the WT. (D) Normalized contact map binned at 5kb of *parEts* pBAD *topB* grown at 30°C. Genome coordinates are indicated by the x and y axes. Interesting positions of the genome are indicated above the plot. *matS* sites are represented as gray bars. Macrodomain are represented by light green (ori), dark green (right), red (ter), blue (left), gray (NR/NL). For the normalized contact maps, the color scale of the frequency of contacts between two regions of the genome is indicated below (arbitrary units), from white (rare contacts) to dark red (frequent contacts). For the ratio matrices, a decrease or increase in contacts in the mutant cells compared with the control is represented with a blue or red color, respectively. White indicates no differences between the two conditions.

Supplementary Figure 8

(A) Normalized contact map binned at 5kb of *matP*, (B) Normalized contact map binned at 5kb of *matP parEts*, (C) Ratio of the normalized contact maps of *parE^{ts} matP* and *parE^{ts}* 1h after shift at 42°C. (D) Ratio of the normalized contact maps of *parE^{ts} matP* and *matP* 1h after shift at 42°C. (E) Normalized contact map binned at 5kb of WT inverted 1h after shift at 42°C. (F) Normalized contact map binned at 5kb of *parEts* inverted 1h after shift at 42°C, (G) On the left, normalized contact map binned at 5kb of *mukB parEts* grown at 30°C and then shifted for 1h at 42°C. In the centre, ratio of the normalized contact maps of *parEts mukB* and *parEts* 1h after shift at 42°C. On the right, ratio of the normalized contact maps of *parEts mukB* and *mukB* 1h after shift at 42°C. For the normalized contact maps, the color scale of the frequency of contacts between two regions of the genome is indicated below (arbitrary units), from white (rare contacts) to dark red (frequent contacts). For the ratio matrices, a decrease or increase in contacts in the mutant cells compared with the control is represented with a blue or red color, respectively. White indicates no differences between the two conditions. H) Flow cytometry analysis of the *parC^{ts} matP, parE^{ts} matP and parE^{ts} mukB* strains grown for 1h at non permissive temperature. DNA content (black). Number of origins after replication run out (red). Data

are directly comparable to the one presented on supplementary Figure 1 for the single *parC*^{ts} and *parE*^{ts} mutants.

Supplementary Figure 9

Intracellular localization of *left 1* and *ter parS*/ParB tags in the *parC*^{ts} *matP* strain after 1h at 30°C. A) Scatter plot of the positioning of the foci along the length of the cell according to cell size. Left panel the two tags are represented, the middle and right panels respectively show the *left1*-ParB$^{P1\text{-}CFP}$ and *ter*-ParB$^{pMT1\text{-}yGFP}$ foci alone. The blue line marks mid-cell. Empty circle (focus 1), plain circle (focus2). B) Scatter plot of the relative distance between *left* and *ter* foci according to the positioning of the left focus. Histograms show the density of points in a given bin of the scatter plot for the *par*^{Cts} strain (Supplementary Figure 4) and the *parC*^{ts} *matP* strain (A).

Supplementary Table 1: Strain used in this work

GENOTYPE	REFERENCE OR SOURCE
MG1655	Espeli lab
MG1655 *gyrBts*	Espeli lab
MG1655 *parEts*::Tc	Espeli lab
MG1655 *parCts*::Tc	Espeli lab
MG1655 topB::cm	Nurse et al 2003(3)
MG1655 *parEts*::Tc *ΔtopB*::Cm	This work
MG1655 *parEts*:TC + pBR-pBAD-*topB*:Kan	Espeli lab
MG1655 LR17 ptsACXI attlR attL (0.806549Mb/1.342702Mb)	Valens et al 2004 (4)
MG1655 inverted *parEts* (0.806549Mb/1.342702Mb)	This work
MG1655 <u>*ΔmatP*</u>	Espeli lab
MG1655 *ΔmatP parEts*:Tc	El Sayyed et al 2016 (5)
MG1655 *ΔmukB*	Espeli lab
MG1655 *ΔmukB parEts* ::Tc	This work
MG59 (MG1555 tos::dif N15)	Cui et al 2007 (6)
MG59 *ΔmatP*	Espeli et al 2012 (7)
MG59 *parEts*	This work
MG59 *ΔmatP parEts*	This work
MG1655 *aidB* parS P1 *gusC* parS *PMT1* pFH2973	Espeli Lab
MG1655 *ygeB* parS P1 *gusC* parS *PMT1* pFH2973	Espeli Lab
MG1655 *ybfD* parS P1 *gusC* parS *PMT1* pFH2973	Espeli Lab
MG1655 *crl* parS P1 *cynX* parS *PMT1* pFH2973	Espeli Lab
MG1655 *gusC* parS P1 *yoaC* parS T1 pFH2973	Espeli Lab
MG1655 *ybfD* parS P1 *yfgE* parS *PMT1* pFH2973	Espeli Lab
MG1655 *parCts aidB* parS P1 *gusC* parS *PMT1* pFH2973	This work
MG1655 *parCts ygeB* parS P1 *gusC* parS *PMT1* pFH2973	This work
MG1655 *parCts ybfD* parS P1 *gusC* parS *PMT1* pFH2973	This work
MG1655 *parCts crl* parS P1 *cynX* parS *PMT1* pFH2973	This work
MG1655 *parCts gusC* parS P1 *yoaC* parS T1 pFH2973	This work
MG1655 *parCts matP ygeB* parS P1 *gusC* parS *PMT1* pFH2973	This work
This work	This work
Salmonella Typhimurium LT2	Gift from Patrick Higgins lab
Salmonella Typhimurium LT2 *parC281*(TS) zge-2393::Tn10	Gift from Patrick Higgins lab
Salmonella Typhimurium LT2 *parE206*(TS) zge-2393::Tn10	Gift from Patrick Higgins lab

KEY RESOURCES TABLE

REAGENT or RESOURCE	SOURCE	IDENTIFIER
Chemicals, Peptides, and Recombinant Proteins		
2ml VK05 microorganism PreCellys tubes	Bertin Instruments	Cat# P000913-LYSK0-A
microTUBE AFA Fiber Pre-Slit Snap-Cap	Covaris	Cat# 520045
100mM dNTPs (set of 4)	Dutscher	Cat# 755086
Proteinase K (20mg/ml)	Eurobio Scientific	Cat# GEXPRK01-B5
Ethidium Bromide Solution	Eurobio Scientific	Cat# GEPBET02-AF
RNase A	Euromedex	Cat# RB0473
HpaII	New England Biolabs	Cat# R0171M
DNA Polymerase I, Large (Klenow) Fragment	New England Biolabs	Cat# M0210L
10x NEBuffer 2	New England Biolabs	Cat# B7002S
Quick Ligation Kit	New England Biolabs	Cat# M2200L
10x T4 DNA Ligase Reaction Buffer	New England Biolabs	Cat# B0202S
Phusion High-Fidelity PCR Master Mix with HF Buffer	New England Biolabs	Cat# M0531L
T4 Polynucleotide Kinase	New England Biolabs	Cat# M0201L
T4 DNA Polymerase	New England Biolabs	Cat# M0203L
Klenow Fragment (3'-5'exo-)	New England Biolabs	Cat# M0212L
36.5 – 38% Formaldehyde solution	Sigma-Aldrich	Cat# F8775
Glycine	Sigma-Aldrich	Cat# G8898
1M MgCl2	Sigma-Aldrich	Cat# M1028
DL-Dithiothreitol solution	Sigma-Aldrich	Cat# 43816
Bovine Serum Albumin	Sigma-Aldrich	Cat# A7906
ATP	Sigma-Aldrich	Cat# 10519987001
Sodium acetate	Sigma-Aldrich	Cat# S2889
Phenol:Chloroform:Isoamyl Alcohol 25:24:1 (pH 8.0)	Sigma-Aldrich	Cat# P2069
cOmplete Mini EDTA-free protease inhibitor cocktail	Sigma-Aldrich	Cat# 11836170001
T4 DNA ligase (30U/ul)	Thermo Fisher Scientific	Cat# EL0013
20% SDS solution	Thermo Fisher Scientific	Cat# 10607633
Triton-X-100	Thermo Fisher Scientific	Cat# 10671652
1M Tris-Hcl pH7.5	Thermo Fisher Scientific	Cat# 10573145
0.5M EDTA (pH 8.0)	Thermo Fisher Scientific	Cat# 10182903
Absolute EtOH	Thermo Fisher Scientific	Cat# 10680993
10X PBS solution	Thermo Fisher Scientific	Cat# 10649743
UltraPure Agarose	Thermo Fisher Scientific	Cat# 16500500
50x TAE Solution	Thermo Fisher Scientific	Cat# 10490264
Biotin-14-dCTP	Thermo Fisher Scientific	Cat# 19518018
NaCl	Thermo Fisher Scientific	Cat# 10616082
Tween 20	Thermo Fisher Scientific	Cat# 10113103
Dynabeads MyOne Streptavidin C1	Thermo Fisher Scientific	Cat# 10202333
GeneRuler 1Kb Plus DNA ladder	Thermo Fisher Scientific	Cat# SM1331
Orange DNA Loading Dye	Thermo Fisher Scientific	Cat# R0631
DL-Serine hydroxamate seryl-tRNA synthetase inhibitor	Sigma-Aldrich	CAS# 55779-32-3
Critical Commercial Assays		
Agencourt Ampure XP beads	Beckman Coulter	Cat# A63881
Qubit dsDNA HS assay kit	Thermo Fisher Scientific	Cat# 10606433

NextSeq 500/550 v2.5 High Output Kit (75 cycles)	Illumina	Cat# 20024906
Truseq DNA CD Indexes (96 indexes)	Illumina	Cat# 20015949
Deposited Data		
Raw data and processed data	This study	PRJNA587586
BC51: Hi-C of WT *E.coli* – exp phase 30°C – HpaII – rep1- unprocessed fastq	This study	
BC52: Hi-C of WT *E.coli* – exp phase 1h at 42°C – HpaII – rep1- unprocessed fastq	This study	
BC53: Hi-C of WT *E.coli parEts*– exp phase 30°C – HpaII – rep1- unprocessed fastq	This study	
BC54: Hi-C of WT *E.coli parEts* – exp phase 1h at 42°C – HpaII – rep1- unprocessed fastq	This study	
BC40: Hi-C of WT *E.coli parEts* – exp phase 1h at 42°C – HpaII – rep1- unprocessed fastq	This study	
BC23: Hi-C of WT *E.coli parEts* – exp phase 1h at 42°C – HpaII – rep1- unprocessed fastq	This study	
BC55: Hi-C of WT *E.coli parEts ΔtopB* – exp phase 1h at 42°C – HpaII – rep1- unprocessed fastq	This study	
BC56: Hi-C of WT *E.coli parEts ΔtopB* – exp phase 30°C – HpaII – rep1- unprocessed fastq	This study	
BC57: Hi-C of WT *E.coli* pBR *parE* – exp phase 30°C – HpaII – rep1- unprocessed fastq	This study	
BC58: Hi-C of WT *E.coli* pBR *parE* – exp phase 1h at 42°C – HpaII – rep1- unprocessed fastq	This study	
BC59: Hi-C of WT *E.coli parEts* pBR *parE* – exp phase 30°C – HpaII – rep1- unprocessed fastq	This study	
BC60: Hi-C of WT *E.coli parEts* pBR *parE* – exp phase 1h at 42°C – HpaII – rep1- unprocessed fastq	This study	
BC61: Hi-C of WT *E.coli ΔtopB* – exp phase 30°C – HpaII – rep1- unprocessed fastq	This study	
BC62: Hi-C of WT *E.coli ΔtopB* – exp phase 1h at 42°C – HpaII – rep1- unprocessed fastq	This study	
BC80: Hi-C of WT linear *E.coli* – exp phase 1h at 42°C – HpaII – rep1- unprocessed fastq	This study	
BC87: Hi-C of WT linear *E.coli parEts* – exp phase 1h at 42°C – HpaII – rep1- unprocessed fastq	This study	
BC82: Hi-C of WT *E.coli ΔmatP* – exp phase 1h at 42°C – HpaII – rep1- unprocessed fastq	This study	
BC83: Hi-C of WT *E.coli ΔmatP parEts* – exp phase 1h at 42°C – HpaII – rep1- unprocessed fastq	This study	
BC84: Hi-C of WT *E.coli* inverted – exp phase 1h at 42°C – HpaII – rep1- unprocessed fastq	This study	
BC85: Hi-C of WT *E.coli parEts* inverted – exp phase 1h at 42°C – HpaII – rep1- unprocessed fastq	This study	
BC88: Hi-C of WT *E.coli* pBAD *ΔtopB* – exp phase 1h at 42°C + arabinose 0.2% – HpaII – rep1- unprocessed fastq	This study	
BC89: Hi-C of WT *E.coli parEts* pBAD *ΔtopB* – exp phase 1h at 42°C + arabinose 0.2% – exp phase 1h at 42°C – HpaII – rep1- unprocessed fastq	This study	
BC90: Hi-C of WT *E.coli parCts* – exp phase 30°C – HpaII – rep1- unprocessed fastq	This study	
BC91: Hi-C of WT *E.coli parCts* – exp phase 1h at 42°C – HpaII – rep1- unprocessed fastq	This study	
BC101: Hi-C of WT *E.coli* – exp phase 30°C + SHX (10mg/ml) – HpaII – rep1- unprocessed fastq	This study	
BC102: Hi-C of WT *E.coli* – exp phase 1h at 42°C + SHX (10mg/ml) – HpaII – rep1- unprocessed fastq	This study	

BC103: Hi-C of WT *E.coli parEts* – exp phase 30°C + SHX (10mg/ml) – HpaII – rep1- unprocessed fastq	This study	
BC104: Hi-C of WT *E.coli parEts* – exp phase 1h at 42°C + SHX (10mg/ml) – HpaII – rep1- unprocessed fastq	This study	
BC109: Hi-C of WT *E.coli* – exp phase 10min after shift at 42°C – HpaII – rep1- unprocessed fastq	This study	
BC110: Hi-C of WT *E.coli* – exp phase 20min after shift at 42°C – HpaII – rep1- unprocessed fastq	This study	
BC111: Hi-C of WT *E.coli* – exp phase 30min after shift at 42°C – HpaII – rep1- unprocessed fastq	This study	
BC112: Hi-C of WT *E.coli* – exp phase 40min after shift at 42°C – HpaII – rep1- unprocessed fastq	This study	
BC113: Hi-C of WT *E.coli* – exp phase 50min after shift at 42°C – HpaII – rep1- unprocessed fastq	This study	
BC114: Hi-C of WT *E.coli parEts* – exp phase 10min after shift at 42°C – HpaII – rep1- unprocessed fastq	This study	
BC115: Hi-C of WT *E.coli parEts* – exp phase 20min after shift at 42°C – HpaII – rep1- unprocessed fastq	This study	
BC116: Hi-C of WT *E.coli parEts* – exp phase 30min after shift at 42°C – HpaII – rep1- unprocessed fastq	This study	
BC117: Hi-C of WT *E.coli parEts* – exp phase 40min after shift at 42°C – HpaII – rep1- unprocessed fastq	This study	
BC118: Hi-C of WT *E.coli parEts* – exp phase 50min after shift at 42°C – HpaII – rep1- unprocessed fastq	This study	
BC119: Hi-C of WT *E.coli* – exp phase 1h at 42°C + rifampicin (100ng/µl) – HpaII – rep1- unprocessed fastq	This study	
BC120: Hi-C of WT *E.coli parEts* – exp phase 1h at 42°C + rifampicin (100ng/µl) – HpaII – rep1- unprocessed fastq	This study	
BC127: Hi-C of WT *E.coli parEts ΔmatP* pBAD *matP* + glucose – exp phase 30min at 42°C – HpaII – rep1- unprocessed fastq	This study	
BC128: Hi-C of WT *E.coli parEts ΔmatP* pBAD *matP* + arabinose – exp phase 30min at 42°C – HpaII – rep1- unprocessed fastq	This study	
BC129: Hi-C of WT *E.coli parEts ΔmukB* – exp phase 25°C – HpaII – rep1- unprocessed fastq	This study	
BC130: Hi-C of WT *E.coli parEts ΔmukB* – exp phase 30°C – HpaII – rep1- unprocessed fastq	This study	
BC131: Hi-C of WT *E.coli parEts ΔmukB* – exp phase 1h at 42°C – HpaII – rep1- unprocessed fastq	This study	
BC132: Hi-C of WT *E.coli* – exp phase 60min after shift at 42°C – HpaII – rep1- unprocessed fastq	This study	
BC133: Hi-C of WT *E.coli parEts* – exp phase 60min after shift at 42°C – HpaII – rep1- unprocessed fastq	This study	
BC134: Hi-C of WT *S.thyphimurium* – exp phase 1h at 42°C – HpaII – rep1- unprocessed fastq	This study	
BC135: Hi-C of WT *S.thyphimurium parEts* – exp phase 1h at 42°C – HpaII – rep1- unprocessed fastq	This study	
BC136: Hi-C of WT *S.thyphimurium parCts* – exp phase 1h at 42°C – HpaII – rep1- unprocessed fastq	This study	
BC139: Hi-C of WT *E.coli* – exp phase 2h at 42°C – HpaII – rep1- unprocessed fastq	This study	
BC140: Hi-C of WT *E.coli parEts* – exp phase 2h at 42°C – HpaII – rep1- unprocessed fastq	This study	
BC142: Hi-C of WT *E.coli Δdif* – exp phase 1h at 42°C – HpaII – rep1- unprocessed fastq	This study	

BC143: Hi-C of WT *E.coli parEts Δdif* – exp phase 1h at 42°C – HpaII – rep1- unprocessed fastq	This study	
Experimental Models: Organisms/Strains		
E. coli K12 MG1655 (*F- lambda- ilvG- rfb-50 rph-1)* : individual genotype see Table S1		
Salmonella Typhimurium LT2 : individual genotype see Table S1	(Hoiseth and Stocker, 1981)	N/A
Software and Algorithms		
Bowtie2 (Langmead and Salzberg, 2012), http://bowtie-bio.sourceforge.net/bowtie2/index.shtml		
SCN normalization procedure	(Cournac et al., 2012)	https://github.com/koszullab/hicstuff
R	(RStudio Team, 2020)	https://www.r-project.org
Other		
Codes and functions for Hi-C analysis https://github.com/koszullab/hicstuff		
