# Supplementary figures and images for: Extended sister-chromosome catenation leads to massive reorganization of the E. coli genome
Source: Nucleic Acids Res. 2022 Feb 25;50(5):2635–50. doi: 10.1093/nar/gkac105 (PMC8934667; doi:10.1093/nar/gkac105)

**A.**

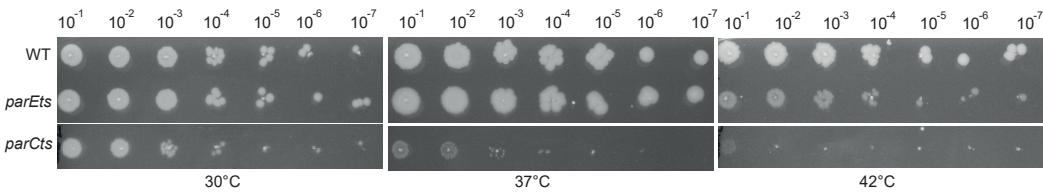

**B.**

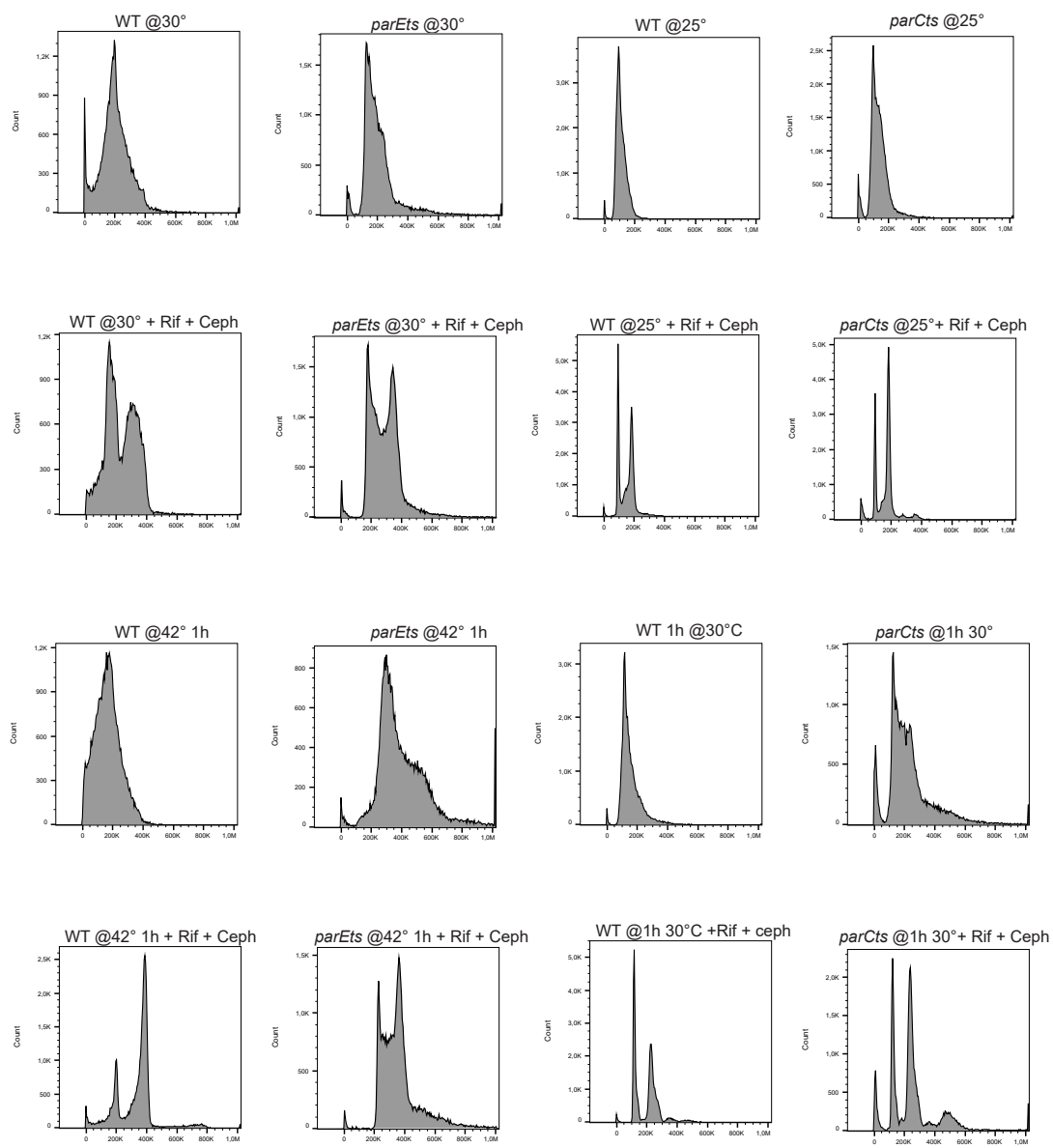

**C.**

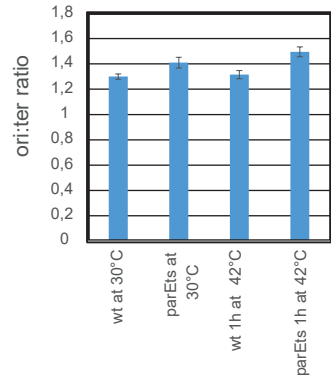

Supplement: gkac105_Supplemental_Files [file gkac105_supplemental_files.zip › supp_fig1_rev_v1.pdf]

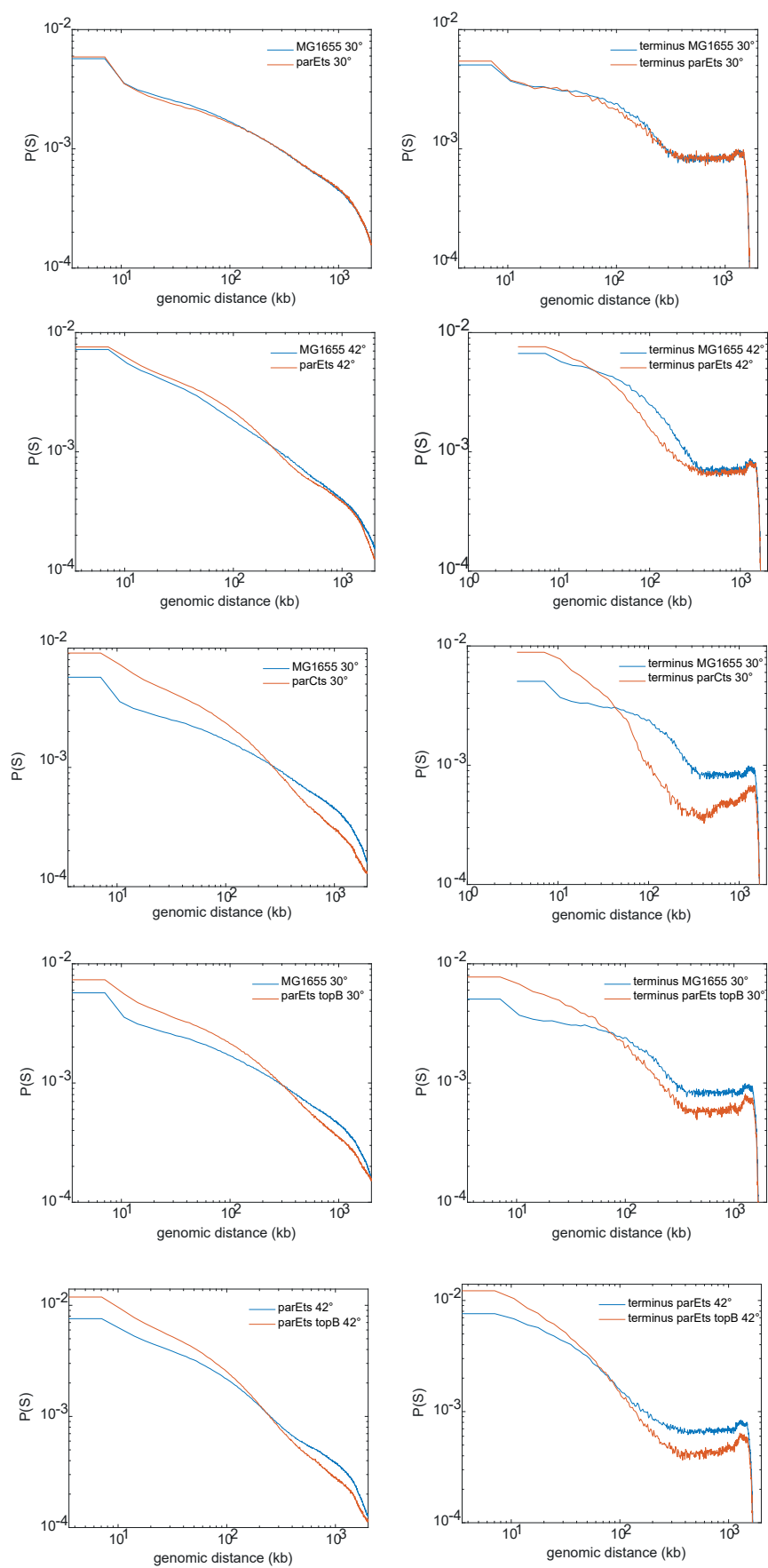

Supplement: gkac105_Supplemental_Files [file gkac105_supplemental_files.zip › supp_fig2_rev_v1.pdf]

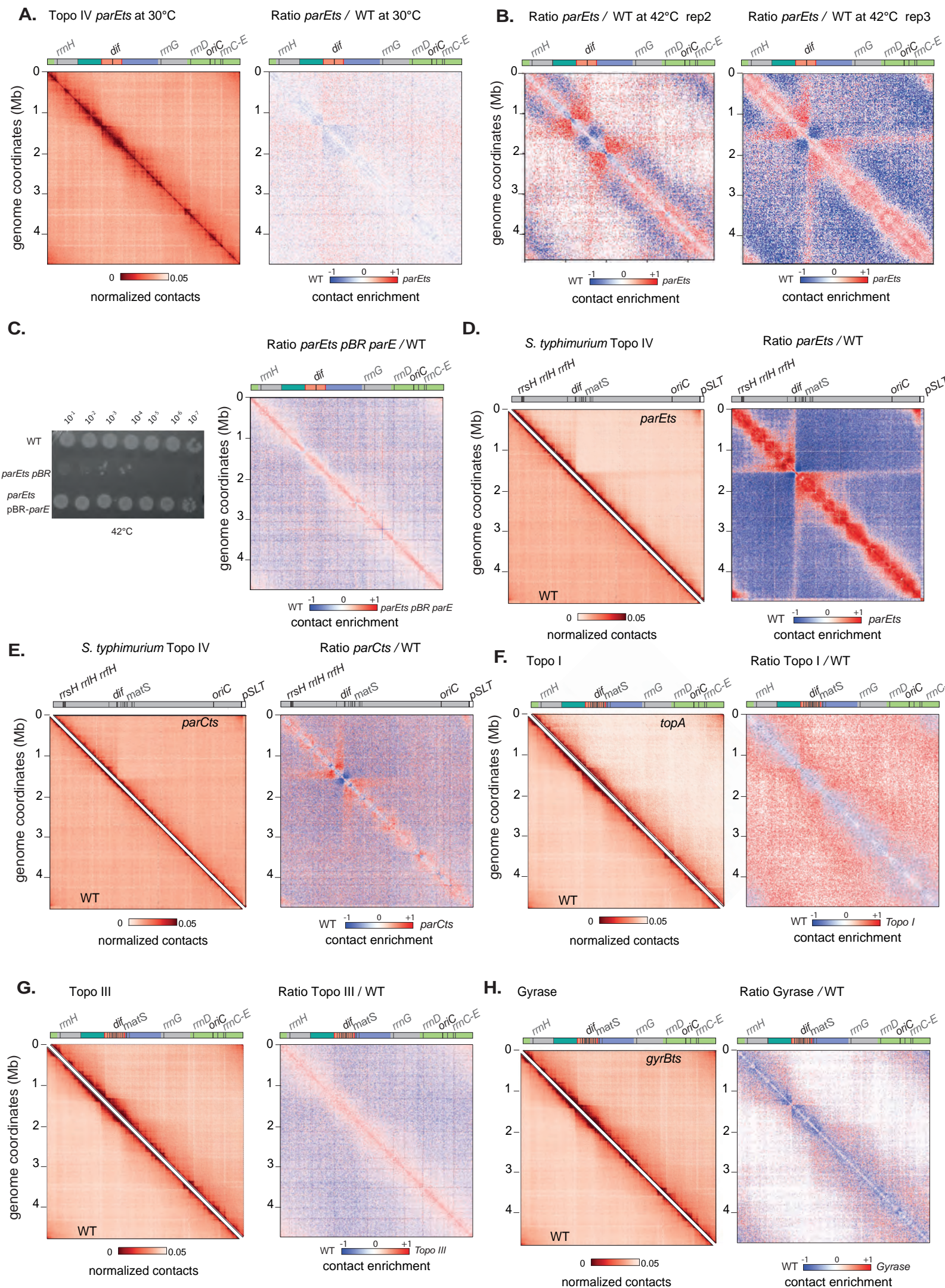

Supplement: gkac105_Supplemental_Files [file gkac105_supplemental_files.zip › supp_fig3_rev_v1_red.pdf]

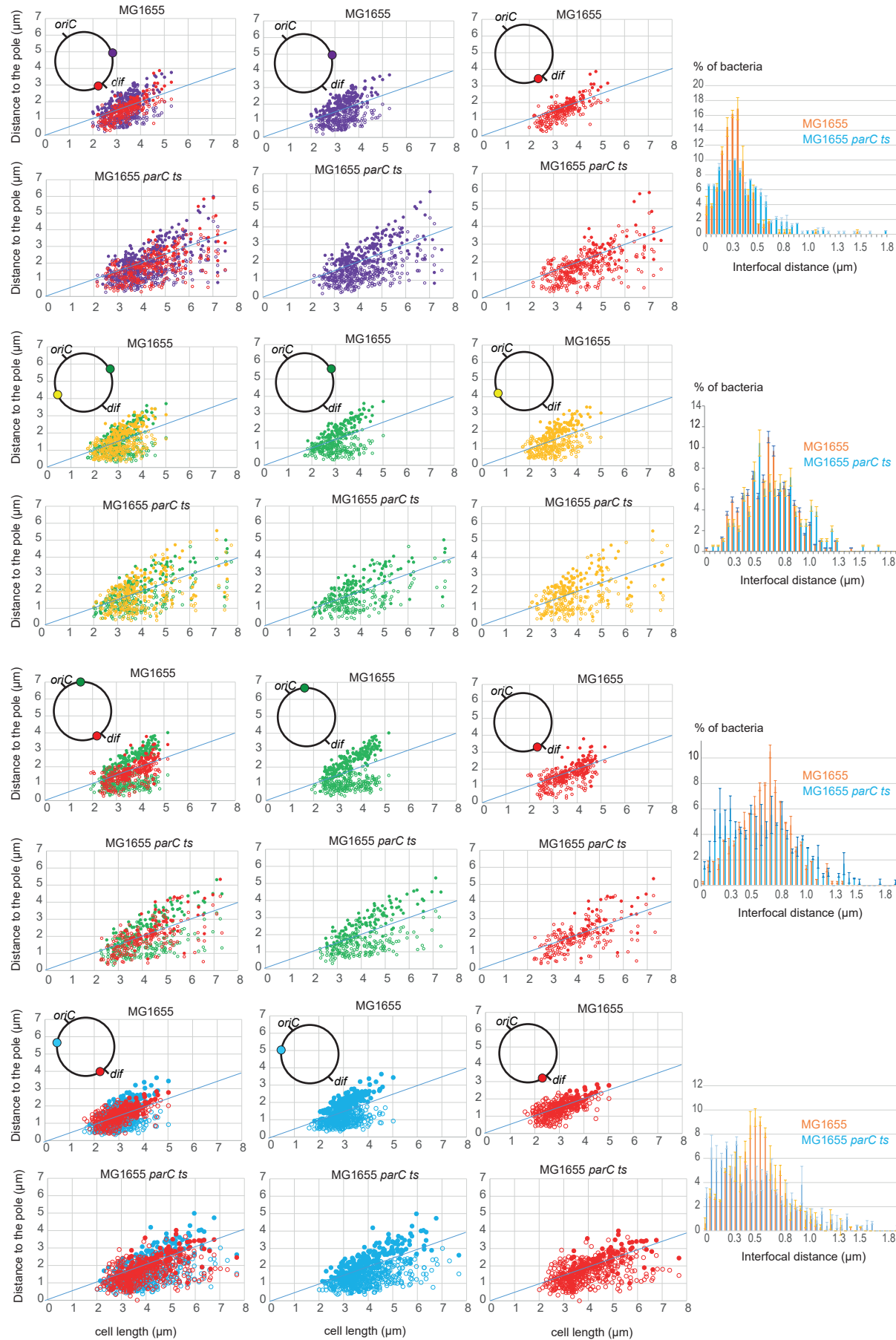

Supplement: gkac105_Supplemental_Files [file gkac105_supplemental_files.zip › supp_fig4_rev_v1.pdf]

A.

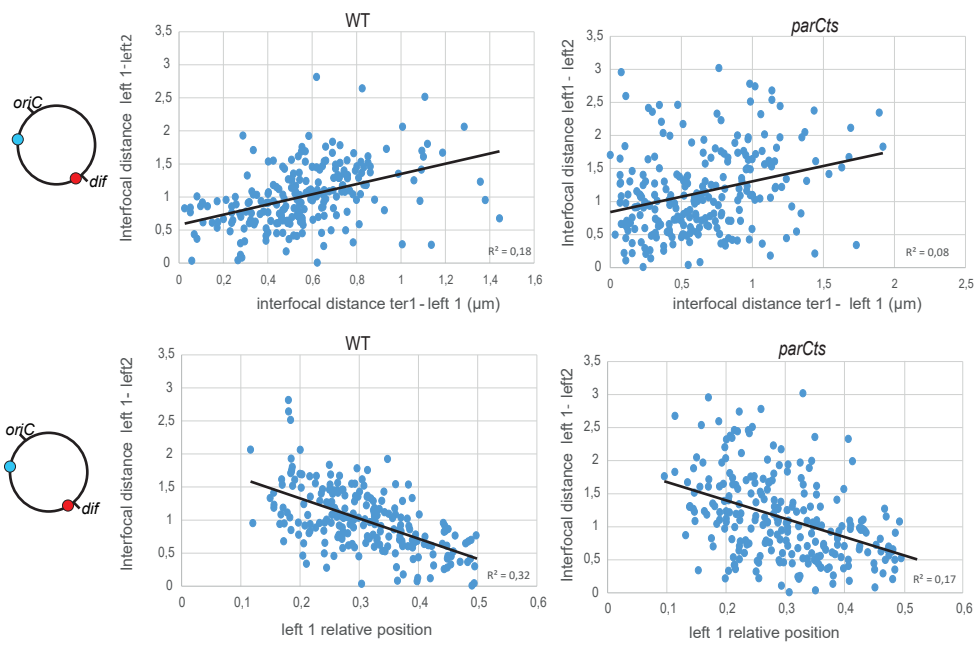

B.

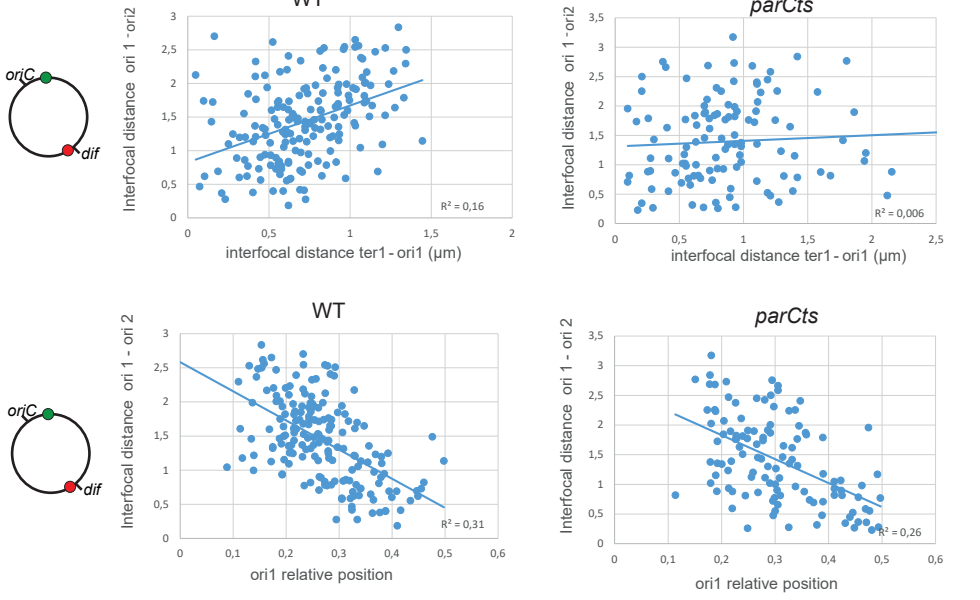

Supplement: gkac105_Supplemental_Files [file gkac105_supplemental_files.zip › supp_fig5_rev_v2.pdf]

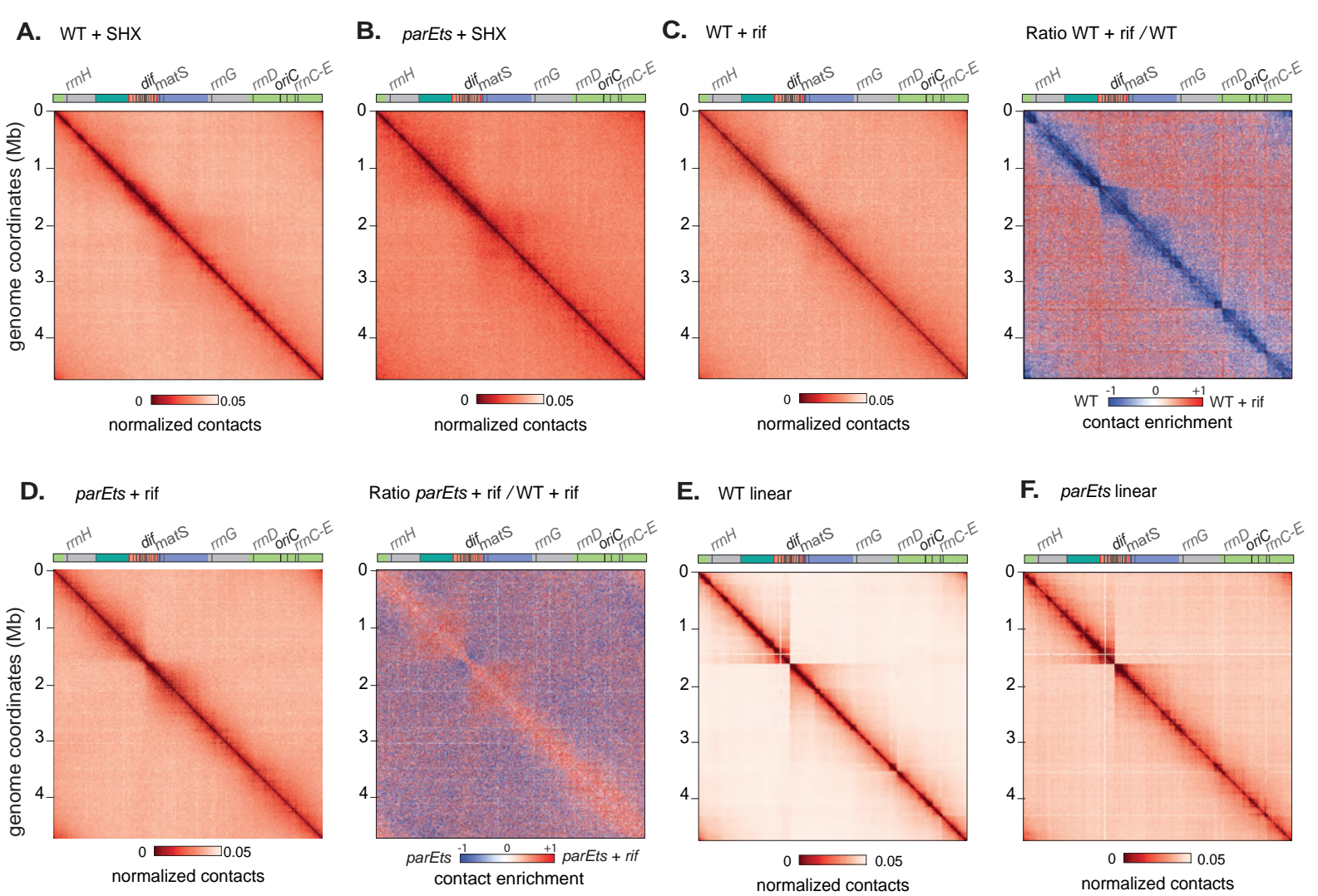

Supplement: gkac105_Supplemental_Files [file gkac105_supplemental_files.zip › supp_fig6_rev_v2_red.pdf]

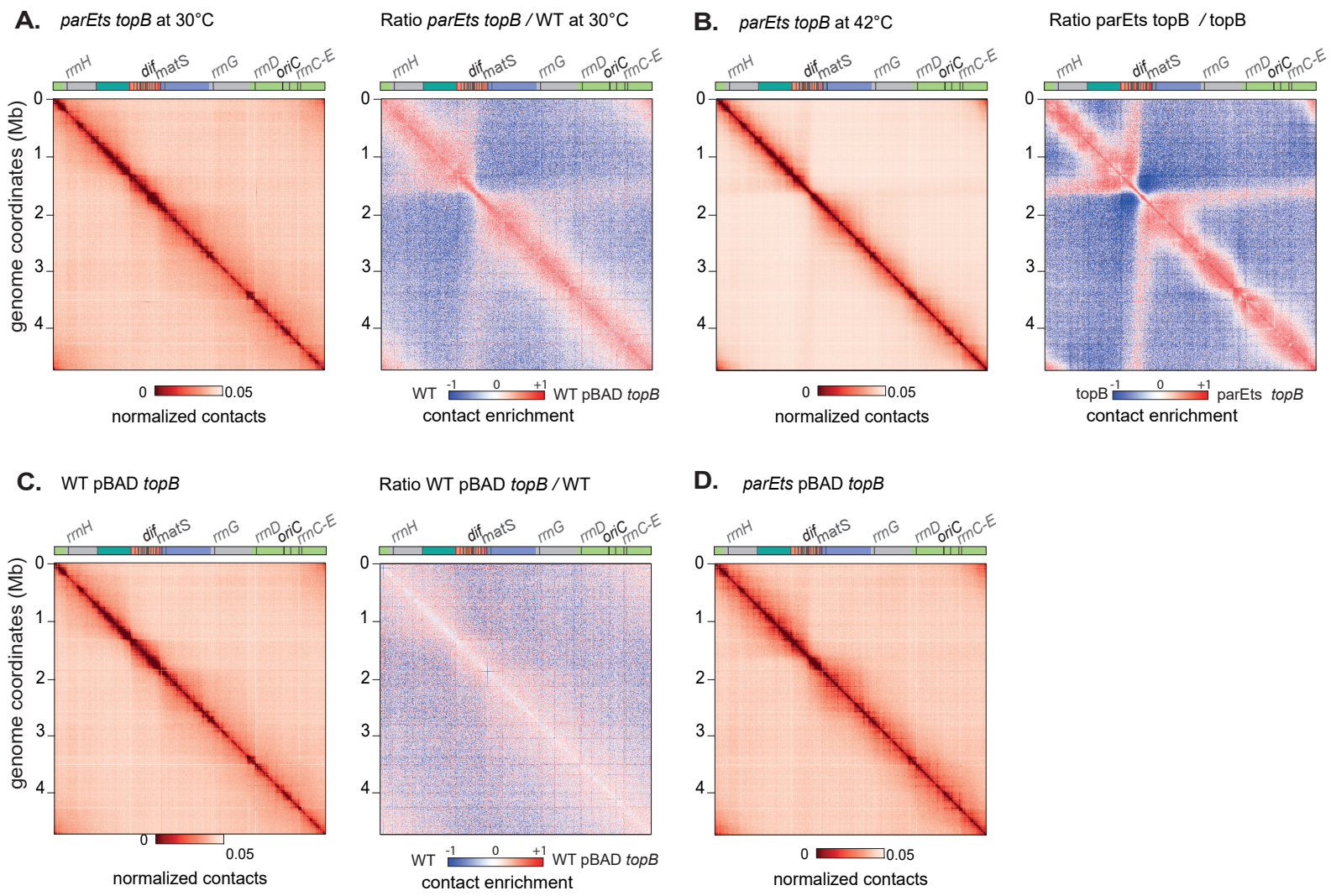

Supplement: gkac105_Supplemental_Files [file gkac105_supplemental_files.zip › supp_fig7_rev_v2.pdf]

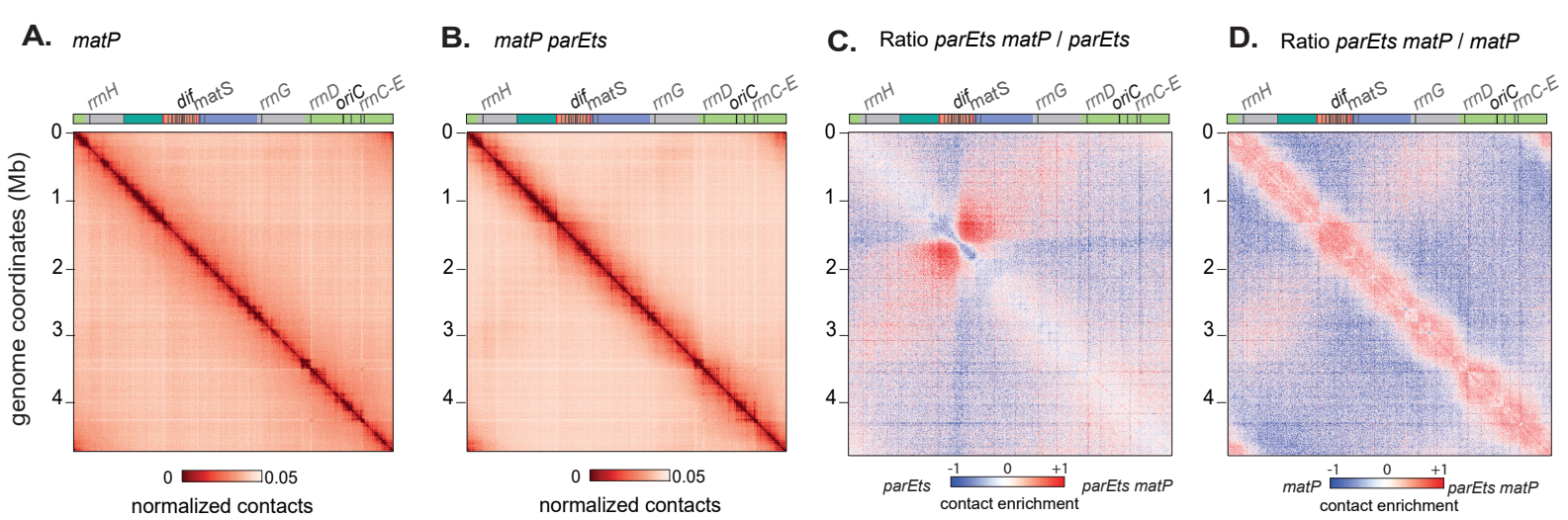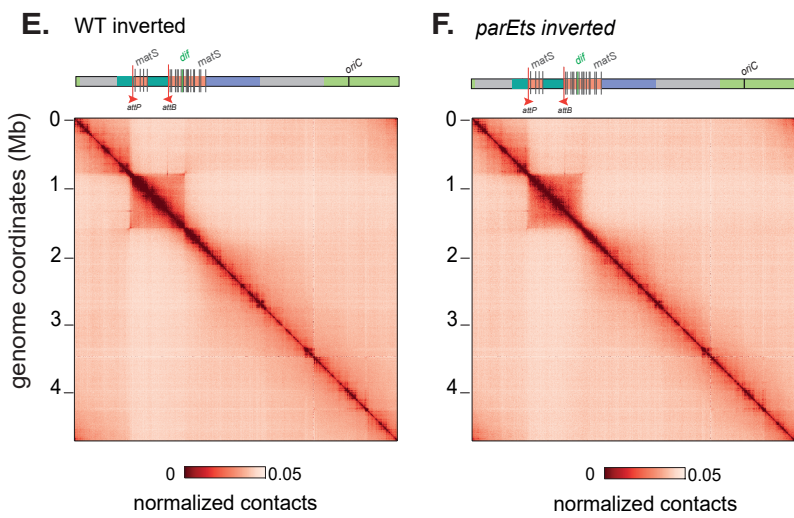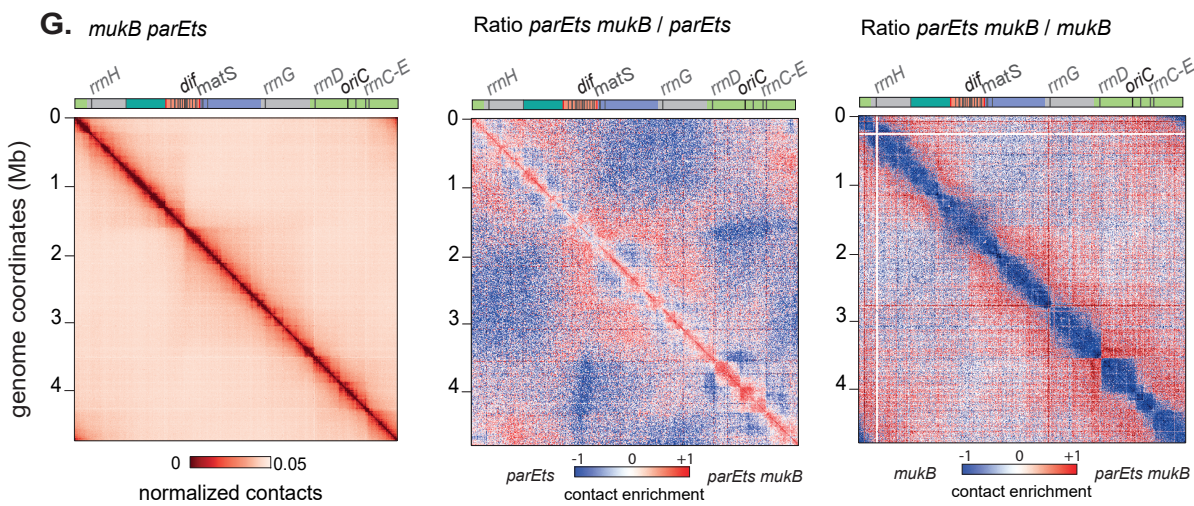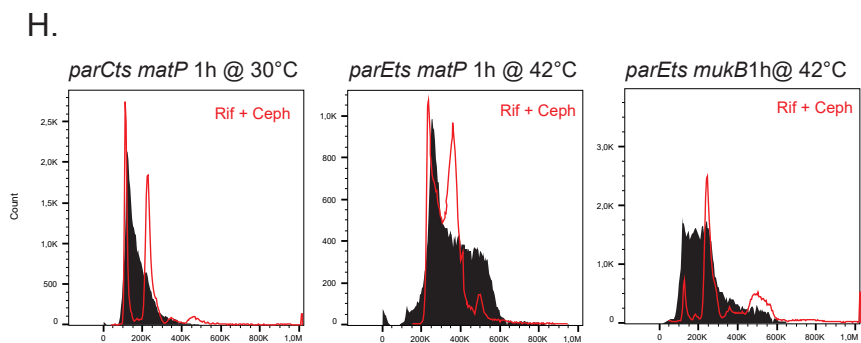

Supplement: gkac105_Supplemental_Files [file gkac105_supplemental_files.zip › supp_fig8_rev_v2.pdf]

A.

MG1655 *parCts matP*

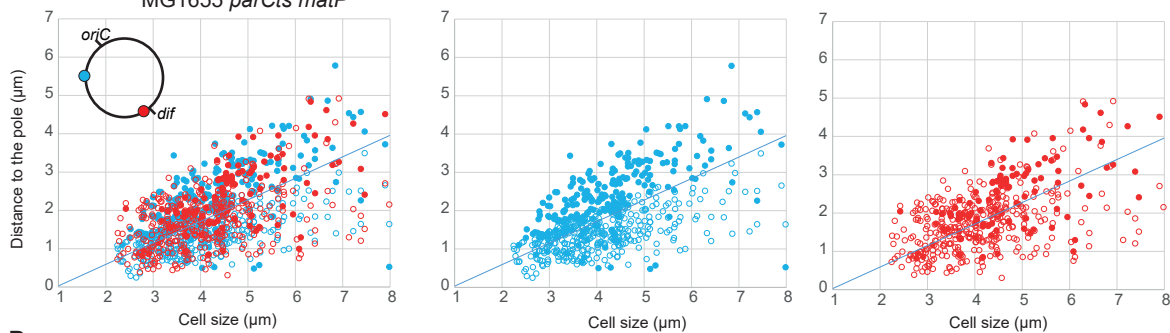

B.

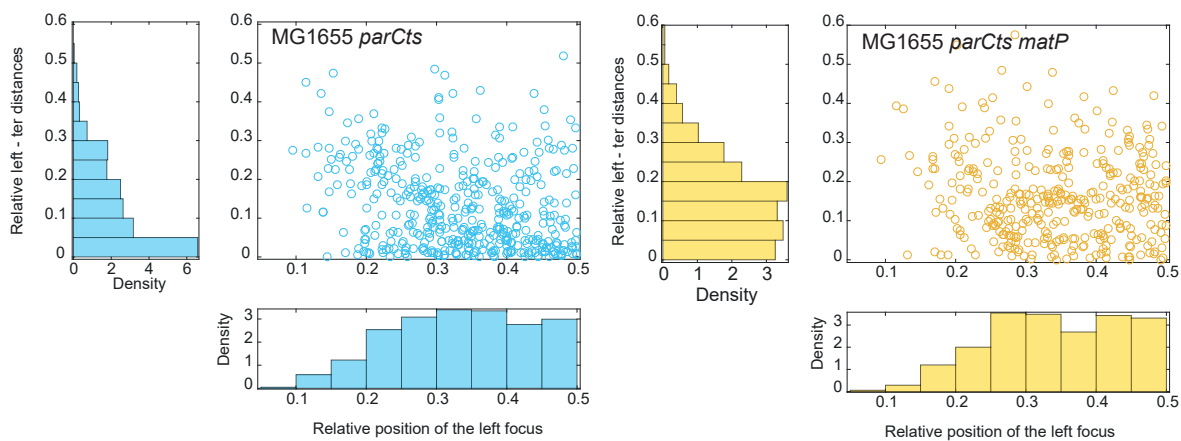

Supplement: gkac105_Supplemental_Files [file gkac105_supplemental_files.zip › supp_fig9_rev_v2.pdf]
